# Motor Activity Dependent and Independent Functions of Myosin II Contribute to Actomyosin Ring Assembly and Contraction in *Schizosaccharomyces pombe*

**DOI:** 10.1016/j.cub.2017.01.028

**Published:** 2017-03-06

**Authors:** Saravanan Palani, Ting Gang Chew, Srinivasan Ramanujam, Anton Kamnev, Shrikant Harne, Bernardo Chapa-y-Lazo, Rebecca Hogg, Mayalagu Sevugan, Mithilesh Mishra, Pananghat Gayathri, Mohan K. Balasubramanian

**Affiliations:** 1 Division of Biomedical Sciences, Warwick Medical School, University of Warwick, Coventry CV4 7AL, UK; 2 School of Biological Sciences, National Institute of Science Education and Research (NISER), Odisha 752050, India; 3 Temasek Life Sciences Laboratory, 1. Research Link, National University of Singapore, Singapore 117604, Singapore; 4 Department of Biological Sciences, Tata Institute of Fundamental Research (TIFR), Mumbai, Maharashtra 400005, India; 5 Biology Division, Indian Institute of Science Education and Research (IISER), Pune, Maharashtra 411008, India

**Keywords:** myosin-II, actomyosin ring, actin, cytokinesis, *S. pombe*

## Abstract

Cytokinesis depends on a contractile actomyosin ring in many eukaryotes [1, 2, 3]. Myosin II is a key component of the actomyosin ring, although whether it functions as a motor or as an actin cross-linker to exert its essential role is disputed [1, 4, 5]. In *Schizosaccharomyces pombe*, the *myo2*-E1 mutation affects the upper 50 kDa sub-domain of the myosin II heavy chain, and cells carrying this lethal mutation are defective in actomyosin ring assembly at the non-permissive temperature [6, 7]. *myo2*-E1 also affects actomyosin ring contraction when rings isolated from permissive temperature-grown cells are incubated with ATP [8]. Here we report isolation of a compensatory suppressor mutation in the lower 50 kDa sub-domain (*myo2*-E1-Sup1) that reverses the inability of *myo2*-E1 to form colonies at the restrictive temperature. *myo2*-E1-Sup1 is capable of assembling normal actomyosin rings, although rings isolated from *myo2*-E1-Sup1 are defective in ATP-dependent contraction in vitro. Furthermore, the product of *myo2*-E1-Sup1 does not translocate actin filaments in motility assays in vitro. Superimposition of *myo2*-E1 and *myo2*-E1-Sup1 on available rigor and blebbistatin-bound myosin II structures suggests that *myo2*-E1-Sup1 may represent a novel actin translocation-defective allele. Actomyosin ring contraction and viability of *myo2*-E1-Sup1 cells depend on the late cytokinetic *S. pombe* myosin II isoform, Myp2p, a non-essential protein that is normally dispensable for actomyosin ring assembly and contraction. Our work reveals that Myo2p may function in two different and essential modes during cytokinesis: a motor activity-independent form that can promote actomyosin ring assembly and a motor activity-dependent form that supports ring contraction.

## Highlights

• In many eukaryotes, cytokinesis requires an actomyosin-based contractile ring
• The role of motor activity of myosin II in cytokinesis is a topic of active debate
• We isolate a new allele of *S. pombe* Myo2, an essential myosin heavy chain
• We show motor activity-dependent and -independent roles for Myo2

## Teaser

Cytokinesis in many eukaryotes requires an actomyosin-based contractile ring. The role of the motor protein Myosin II in cytokinesis is actively debated. Palani et al. identify a new motor activity-defective allele of fission yeast myosin II and report that the motor activity is dispensable for ring assembly but is essential for ring contraction.

## Results and Discussion

**Figure 1:**
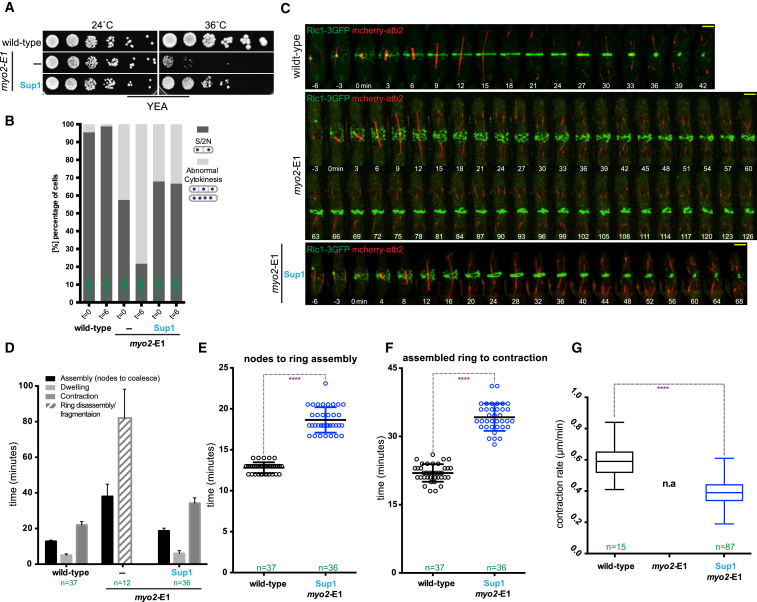
*myo2*-E1-Sup1 Restores Actomyosin Ring Assembly and Partial Ring Contraction (A) Serial dilutions (10-fold) of wild-type, *myo2*-E1, and the intragenic suppressor *myo2*-E1-Sup1 were spotted onto yeast extract agar (YEA) plates and grown for 3 days at 24°C and 36°C. (B) Quantification of DAPI and anillin blue staining used to visualize the nucleus and septum of wild-type, *myo2*-E1, and *myo2*-E1-Sup1 cells, respectively. Phenotypes of the mutants were categorized into two types: septa with two nuclei (S/2N) and cells with abnormal cytokinesis, revealed by the presence of multiple septa and nuclei (MS/>2N). (C) Time-lapse series of wild-type, *myo2*-E1, and *myo2*-E1-Sup1 cells expressing 3GFP-tagged myosin regulatory light chain (Rlc1-3GFP) as a contractile ring marker and mCherry-tagged tubulin (mCherry-atb2) as a cell-cycle stage marker. Cells were grown at 24°C and shifted to 36°C for 3–4 hr before imaging at 36°C (t = 0 indicates the time before Rlc1-3GFP nodes localize to the cell middle). Images shown are maximum-intensity projections of z stacks. Scale bars represent 3 μm. (D) Timing of contractile ring assembly, maturation/dwelling, and contraction. Quantification of (C) is shown. Error bars represent SD. (E) Timing of actomyosin ring assembly from nodes. Quantification of (C) is shown (asterisks indicate the statistical significance of the difference between the two genotypes). Statistical significance was calculated by Student’s t test (^∗∗∗∗^p < 0.0001). Error bars represent SD. (F) Timing of actomyosin ring contraction. Quantification of (C) is shown. Statistical significance was calculated by Student’s t test (^∗∗∗∗^p < 0.0001). Error bars represent SD. (G) Constriction rate determined from a graph of ring circumference versus time. Statistical significance was calculated by Student’s t test (^∗∗∗∗^p < 0.0001). Error bars represent SD. See also Figure S1.

The product of the *myo2*-E1 allele is predicted to harbor a substitution of glycine at position 345 with arginine (Figures S1A and S1B). Cells carrying this mutant allele are capable of colony formation at 24°C but are severely compromised for colony formation at 36°C (Figure 1A) due to defective actomyosin ring assembly [6, 7, 9, 10]. The *myo2*-E1 mutation resides between α-helix HL and β sheet S1D, which is part of the upper 50 kDa sub-domain in the head of Myo2p (Figure S1B). Previous work has shown that *Myo2*-E1p (product of myo2-E1) does not bind or move actin filaments and has a very low ATPase activity in vitro [10, 11]. The presence of a bulky arginine side chain between helices HL and HO in the upper 50 kDa sub-domain of this mutant might introduce constraints to the conformational changes in the Myo2p head domain during the actomyosin cycle, resulting in the observed phenotypes. To further understand the role of Myo2p in cytokinesis, we isolated genetic suppressors that restored the ability of *myo2*-E1 cells to form colonies at 36°C (Figure 1A). One suppressor, *myo2*-E1-Sup1, is described in this study. Genetic crosses between *myo2*-E1-Sup1 and wild-type cells only produced progeny that were able to form colonies at 36°C, suggesting that the suppressor mutation was intragenic or very tightly linked to *myo2*. Nucleotide sequence determination revealed that *myo2*-E1-Sup1 contained the original G345R mutation and also had additional mutations (Q640H and F641I) (Figures S1A and S1B). Furthermore, no sequence alterations were found in the neighboring *rgf3* gene (data not shown), which has also been implicated in cytokinesis [12, 13]. Therefore, we concluded that the sequence alteration Q640H F641I was responsible for the suppression of *myo2*-E1. Interestingly, Q640H and F641I are located in the HW region of the Myo2p head (within the lower 50 KDa sub-domain), which is at a significant distance (∼36 Å) from HL and S1D, the region where the original mutation resides, suggesting potential allosteric mechanisms, rather than a simple reversal of original mutation, may operate in the suppression.

Following a 6 hr shift to 36°C, nearly 80% of *myo2*-E1 cells became multinucleate and had either improper septa with a wavy and patchy appearance or did not have a septum (Figure 1B). By contrast, only ∼35% of *myo2*-E1-Sup1 cells contained such defects, while those defects were rarely seen in wild-type cells (Figures 1B and S1C). Since the ingressing actomyosin ring guides division septum assembly, we investigated the dynamics of the actomyosin ring component Rlc1p-3GFP in wild-type, *myo2*-E1, and *myo2*-E1-Sup1 strains; mCherry-tubulin served as a cell-cycle marker in these experiments. In wild-type cells, actomyosin rings were assembled in metaphase/anaphase A in ∼12.8 ± 0.6 min and contracted following spindle breakdown in ∼22 ± 1.9 min, with an intervening dwell phase of 5 ± 0.8 min during which the actomyosin ring was stably maintained (Figures 1C–1E and S1D). As expected, all aspects of cytokinesis were slower in myo2-E1 mutants compared to wild-type cells: improper ring assembly took ∼38 ± 6.9 min and improper contraction/disassembly lasted ∼82 ± 16.2 min at 36°C (Figures 1C and S1D). Imaging *myo2*-E1-Sup1 cells revealed that they assembled actomyosin rings of normal appearance (Figure 1C, time point 24 min, ending on views in Figure S1D), with a significantly accelerated kinetics for both ring assembly (∼18.6 ± 1.5 min) and contraction (∼34.2 ± 3 min) compared to the original *myo2*-E1 mutant. Nevertheless, both steps were marginally slower in *myo2*-E1-Sup1 compared to wild-type cells (Figures 1C–1E and S1D). Whereas actomyosin rings in wild-type cells contracted at ∼0.6 ± 0.1 μm/min, contraction rate in *myo2*-E1-Sup1 cells was ∼0.4 ± 0.08 μm/min at 36°C. These experiments established that *myo2*-E1-Sup1 assembled contractile rings of normal appearance, although both ring assembly and ring contraction took ∼1.5 times longer compared to wild-type cells.

**Figure 2:**
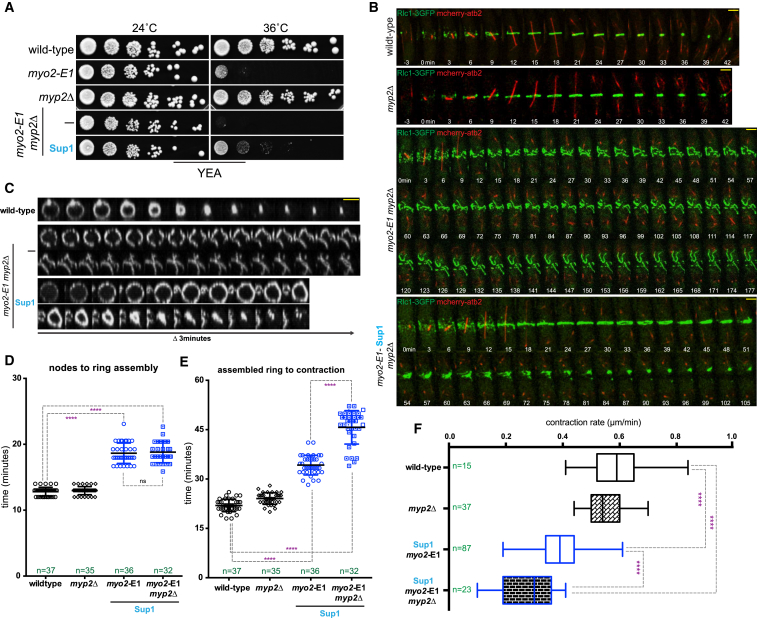
*myo2*-E1-Sup1 Fails in Actomyosin Ring Contraction in the Absence of the Non-essential Myosin Heavy Chain Myp2p (A) Serial dilutions (10-fold) of wild-type, *myo2*-E1, *myp2*Δ, *myo2*-E1 *myp2*Δ, and *myo2*-E1-Sup1 *myp2*Δ were spotted onto YEA plates and grown for 3 days at 24°C and 36°C. (B) Time-lapse series of wild-type, *myp2*Δ, *myo2*-E1 *myp2*Δ, and *myo2*-E1-Sup1 *myp2*Δ cells expressing 3GFP-tagged myosin regulatory light chain (Rlc1-3GFP) as a contractile ring marker and mCherry-tagged tubulin (atb2-mCherry) as a cell-cycle stage marker. Cells were grown at 24°C and shifted to 36°C for 3–4 hr before imaging at 36°C (t = 0 indicates the time before Rlc1-3GFP nodes localize to the cell middle). Images shown are maximum-intensity projections of z stacks. Scale bars represent 3 μm. (C) Kymographs of a 3D-projected ring from wild-type, *myo2*-E1 *myp2*Δ, and *myo2*-E1-Sup1 *myp2*Δ cells. Scale bars represent 3 μm. (D) Timing of actomyosin ring assembly from nodes. Quantification of (B) is shown. Asterisks indicate the statistical significance of the difference between the different genotypes compared to the wild-type. Statistical significance was calculated by Student’s t test (^∗∗∗∗^p < 0.0001). Error bars represent SD. (E) Timing of actomyosin ring contraction. Quantification of Figure 1C and (B) is shown. Statistical significance was calculated by Student’s t test (^∗∗∗∗^p < 0.0001). Error bars represent SD. (F) Constriction rate determined from a graph of ring circumference versus time. Contraction rates of Figure 1C and (B) are shown. Statistical significance was calculated by Student’s t test (^∗∗∗∗^p < 0.0001). Error bars represent SD. See also Figure S2.

Two type II myosin heavy chains participate in cytokinesis in *S. pombe* [14, 15, 16, 17]. We therefore investigated the possibility that Myp2p, which is normally non-essential for ring assembly, assisted in actomyosin ring assembly and contraction in the *myo2*-E1-Sup1 strain through a potential ectopic upregulation. Toward this goal, we generated a double mutant of the genotype *myo2*-E1-Sup1 myp2Δ. Although this strain was viable at 24°C, surprisingly, it was inviable at 36°C (Figure 2A). Time-lapse microscopy was performed on wild-type, *myo2*-E1 *myp2*Δ, *myo2*-E1-Sup1 *myp2*Δ, and *myp2*Δ strains to investigate aspects of actomyosin ring function. The time taken for ring assembly and contraction and the ring contraction rate were comparable in wild-type and *myp2*Δ cells (Figures 2B–2F), clarifying that Myp2p is not important for either ring assembly or contraction at 36°C when Myo2p is fully functional. *myo2*-E1 *myp2*Δ assembled abnormal actomyosin rings that underwent abnormal disassembly (Figures 2B and 2C). *myo2*-E1-Sup1 *myp2*Δ assembled actomyosin rings of normal appearance, and the assembly of these rings took ∼6 min more than wild-type and *myp2*Δ cells (Figures 2B–2D). Ring contraction was dramatically affected in *myo2*-E1-Sup1 *myp2*Δ (Figures 2B, 2C, 2E, and 2F). Contraction and disassembly took more than twice the amount of time compared to wild-type cells, while the ring contraction rate was less than half of that observed in wild-type cells (Figures 2E and 2F). Furthermore, contraction was frequently asymmetric and led to rings disassembling abnormally and often to the fragmentation of the ring into two or more clusters (Figures 2B, time points 48–72 min, and 2C). Since *myo2*-E1-Sup1 *myp2*Δ and *myo2*-E1-Sup1 were capable of actomyosin ring assembly but showed appreciable defects in ring contraction, we conclude Myo2p activity is essential for ring assembly and contraction, whereas Myp2p plays an ancillary role in promoting inefficient contraction when Myo2p motor activity is compromised at 36°C (compare ring contraction times and rates between *myo2*-E1-Sup1 and *myo2*-E1-Sup1 *myp2*Δ in Figures 2E and 2F).

Analysis of three-dimensional structures of rigor myosin (actin bound: 4A7F) and blebbistatin-bound myosin (actin unbound: 1YV3) suggested that the amino acid substitutions in *myo2*-E1-Sup1 may result in increased binding affinity toward F-actin (Figure S2; see the Supplemental Experimental Procedures for a detailed description of the structural analysis). This in turn may lead to defective actomyosin ring contraction due to *myo2*-E1-Sup1 being tightly bound to actin, leading to an actin filament translocation defect.

**Figure 3:**
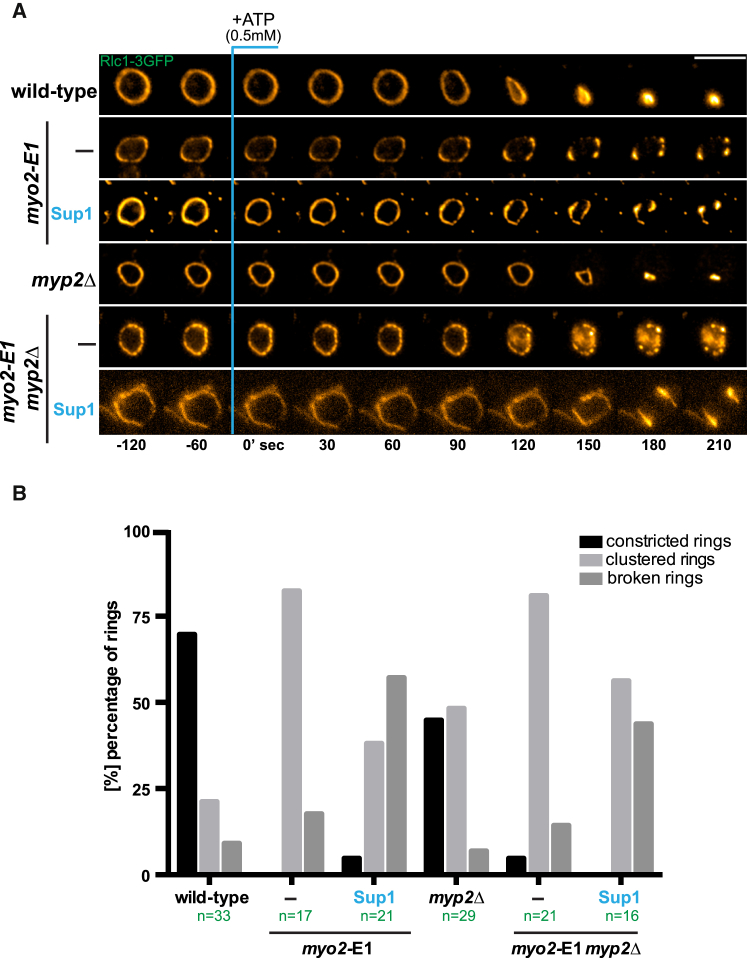
Isolated Actomyosin Rings of *myo2*-E1-Sup1 Do Not Undergo ATP-Dependent Contraction (A) Cell ghosts were prepared from wild-type, *myo2*-E1, *myp2Δ, myo2*-E1 *myp2Δ*, *myo2*-E1-Sup1, and *myo2*-E1-Sup1 *myp2*Δ grown at 24°C. Ring contraction experiments were performed at 24°C and contraction was activated by the addition of 0.5 mM ATP. Images shown are maximum-intensity projections of z stacks. Scale bars represent 5 μm. (B) Graph showing percentage of contracted, clustered, and broken rings. Quantification of (A) is shown. See also Figure S2.

We have already developed methods to isolate ATP-dependent contraction-competent actomyosin rings [8, 18]. We therefore used this system to test if isolated actomyosin rings in cell ghosts from *myo2*-E1-Sup1 were capable of ATP-dependent contraction. Actomyosin rings were isolated from wild-type, *myo2*-E1, *myp2*Δ, *myo2*-E1 *myp2*Δ, *myo2*-E1-Sup1, and *myo2*-E1-Sup1 *myp2*Δ cells grown at the permissive temperature of 24°C. Actomyosin rings isolated from wild-type and myp2Δ cells underwent normal and rapid contraction upon ATP addition (Figures 3A and 3B). As previously reported [8], upon the addition of 0.5 mM ATP, actomyosin rings isolated from *myo2*-E1 and *myo2*-E1 myp2Δ either contracted slowly or underwent fragmentation (Figures 3A and 3B). Interestingly, despite the moderate delay in ring assembly timing, actomyosin rings of normal appearance assembled in *myo2*-E1-Sup1 and *myo2*-E1-Sup1 *myp2*Δ at the restrictive temperature. However, rings isolated from these strains did not contract normally, even at the permissive temperature for *myo2*-E1 (24°C). Instead, rings from these strains remained stable and broke into large fragments. These experiments established that, consistent with in vivo results, rings isolated from *myo2*-E1-Sup1 and *myo2*-E1-Sup1 *myp2*Δ cells are defective in ATP-dependent contraction in vitro. These results were consistent with the idea that the product of *myo2*-E1-Sup1 is defective in its motor activity and actin filament translocation, but not in actin filament binding, which in turn may explain the ability of *myo2*-E1-Sup1 to support actomyosin ring assembly, but not contraction. However, it was possible that the actin translocation defect in *myo2*-E1-Sup1 was due to allosteric effects on other unidentified components of the actomyosin ring that affect ring contraction, rather than a direct effect of *myo2*-E1-Sup1 on actin filament translocation.

**Figure 4:**
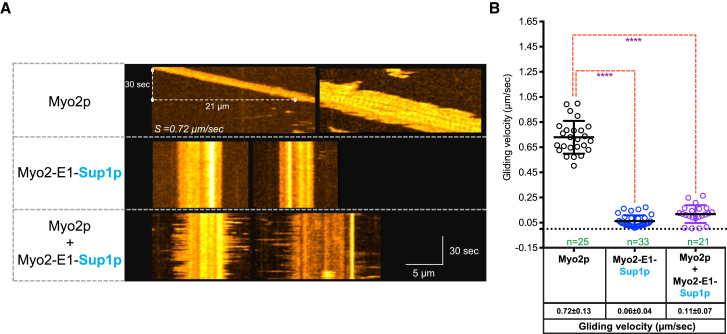
Myo2-E1-Sup1p Showed Tighter Actin Binding but No Motility (A) Type II Myosin-based actin filament-gliding assay. Representative kymographs of time-lapse fluorescence micrographs of actin filaments labeled with rhodamine-phalloidin are shown. Scale bars represent 5 μm. (B) Quantification of the actin filament-gliding assay of (A). Different myosins (Myo2p, Myo2-E1-Sup1p, and Myo2p + Myo2-E1-Sup1p) were tested for gliding velocity (μm/s) using rhodamine-phalloidin-labeled actin. See also Figure S3 and Movies S1, S2, S3, and S4.

To distinguish between these possibilities, we purified the products of *myo2*^+^, *myo2*-E1, and *myo2*-E1-Sup1 using an expression system developed by Lord and Pollard [11]. *Myo2*-E1-Sup1p was more difficult to purify (potentially due to its tight binding to actin) and was eventually isolated from Latrunculin A-treated cells (Figure S3A). We then performed actin motility assays as described in Lord and Pollard [11]. In brief, Myo2p and the mutant versions were immobilized on nitrocellulose-coated coverslips, overlaid with rhodamine-phalloidin-stabilized rabbit actin filaments, and incubated with ATP (Figures 4A, 4B, S3B, and S3C; Movies S1, S2, S3, and S4). We found that wild-type Myo2p was able to bind and translocate actin filaments at ∼0.72 ± 0.13 μm/s when incubated with ATP. Also, as previously reported [11], Myo2-E1p did not attach to actin filaments (Movie S2). Interestingly, unlike the product of *myo2*-E1, the product of *myo2*-E1-Sup1 bound actin tightly, since these filaments were either severely affected for motility or were non-motile (gliding velocity was ∼0.06 ± 0.04 μm/s). Myo2-E1-Sup1p also had a dominant effect when mixed with wild-type Myo2p. The mixture bound to actin filaments but these filaments were non-motile. The fact that Myo2-E1-Sup1p did not support motility, despite binding actin filaments and its dominant-negative effect on motility over wild-type Myo2p, suggests that Myo2-E1-Sup1p is most likely a novel rigor mutant of Myo2p.

Our work reported in this study establishes that the type II myosin, Myo2p, plays two distinct and essential roles. Since cells harboring the novel rigor mutant allele *myo2*-E1-Sup1 assemble normal actomyosin rings, despite the defective contraction in vitro and in vivo, it is possible that actomyosin ring assembly depends on the ability of Myo2p to cross-link actin filaments. Actomyosin ring assembly in *myo2*-E1-Sup1 cells is slower than in wild-type cells (possibly due to cross-linking and tighter binding of Myo2-E1-Sup1p with actin), suggesting that myosin II motor activity may also play a role in actomyosin ring assembly, as previously proposed [19, 20]. It is possible that clustering of cytokinetic precursor nodes can occur through tension generated by myosin II-dependent cross-linking of actin filaments. This view is consistent with aspects of the work of Ma and colleagues who have proposed that actin translocation activity of myosin II is not essential for cytokinesis [4]. Inconsistent with the work of Ma and colleagues, however, are our findings that actomyosin rings in *myo2*-E1-Sup1 cells do not contract normally, that actomyosin rings isolated from those cells fail to undergo ATP-dependent contraction, and that one-step-purified Myo2-E1-Sup1p does not support ATP-dependent actin filament motility in vitro. These observations suggest that myosin II motor activity is essential for actomyosin ring contraction.

Thus, through the analysis of novel myosin II mutant alleles, we have been able to discriminate between myosin II motor activity-dependent and -independent steps in cytokinesis. Published work in *S. cerevisiae* and mammalian cells [4, 5, 21] has questioned the role of myosin II motor activity in cytokinesis. It is likely that in some cell types, tension generated by actin filament cross-linking and filament disassembly alone may suffice for cytokinesis, whereas in others such as *S. pombe*, cytokinesis may depend on motor activity-dependent and -independent functions of myosin II.

## Author Contributions

S.P. conceived and designed experiments, acquired data, performed analysis and interpretation of data, and drafted/revised the article. S.R. and M.M. generated yeast strains and performed preliminary analysis. T.G.C., A.K., S.H., B.C.L., M.S., and R.H. performed analysis and interpretation of data and generated yeast strains and reagents. P.G. performed structural analysis and interpretation of data and drafted/revised the article. M.K.B. conceived the project, conceived and designed experiments, and performed analysis and interpretation of data. S.P. and M.K.B. wrote the manuscript. All authors reviewed the manuscript.

## Supplemental Information

Document S1. Supplemental Experimental Procedures and Figures S1–S3

Movie S1. Filament-Gliding Assay of One-Step-Purified Crude Wild-Type Myosin Myo2p/Cdc4p/Rlc1p Complex, Related to Figure 4 Time-lapse movie of polymerized rhodamine-phalloidin labelled rabbit-skeletal muscle actin filaments in the presence of crude wild-type Myo2p and its associated light chains. Gliding assay was performed as described by Tang et al. (2016) [S5].

Movie S2. Product of Motor-Defective Allele of *myo2*, Myo2-E1p, Did Not Bind Actin Filaments, Related to Figure 4 The product of *myo2*-E1 and its associated light chains (Myo2-E1p/Cdc4p/Rlc1p) was purified at 24°C and the gliding assay was performed as described by Tang et al. (2016) [S5]. Rhodamine-phalloidin labelled rabbit-skeletal muscle actin filaments did not bind to Myo2-E1p.

Movie S3. Myo2-E1p-Sup1 Bound, but Did Not Move, Actin Filaments, Related to Figure 4 The product of *myo2*-E1-Sup1 and its associated light chains (Myo2-E1p/Cdc4p/Rlc1p) was purified at 24°C and the gliding assay was performed as described by Tang et al. (2016) [S5]. Rhodamine-phalloidin labelled rabbit-skeletal muscle actin filaments was found to bind tightly to Myo2-E1p-Sup1 and its associated light chains, but these filaments were not translocated by Myo2-E1-Sup1p.

Movie S4. A Mixture of Myo2p and Myo2-E1p-Sup1p Did Not Support Actin Motility, Related to Figure 4 Pre-mixed 1:1 myosin complex (Myo2p::Myo2-E1p-Sup1) was found to bind tightly to rhodamine-phalloidin labelled actin filaments but fail to move the filaments.

Document S2. Article plus Supplemental Information
