# Supplementary material for: Motor Activity Dependent and Independent Functions of Myosin II Contribute to Actomyosin Ring Assembly and Contraction in Schizosaccharomyces pombe
Source: Curr Biol. 2017 Mar 6;27(5):751–7. doi: 10.1016/j.cub.2017.01.028 (PMC5344676; doi:10.1016/j.cub.2017.01.028)
Supplement: Document S1. Supplemental Experimental Procedures and Figures S1–S3 [file mmc1.pdf]

Current Biology, Volume *27*

Supplemental Information

Motor Activity Dependent and Independent Functions of Myosin II Contribute to Actomyosin Ring Assembly and Contraction in *Schizosaccharomyces pombe*

Saravanan Palani, Ting Gang Chew, Srinivasan Ramanujam, Anton Kamnev, Shrikant Harne, Bernardo Chapa-y-Lazo, Rebecca Hogg, Mayalagu Sevugan, Mithilesh Mishra, Pananghat Gayathri, and Mohan K. Balasubramanian

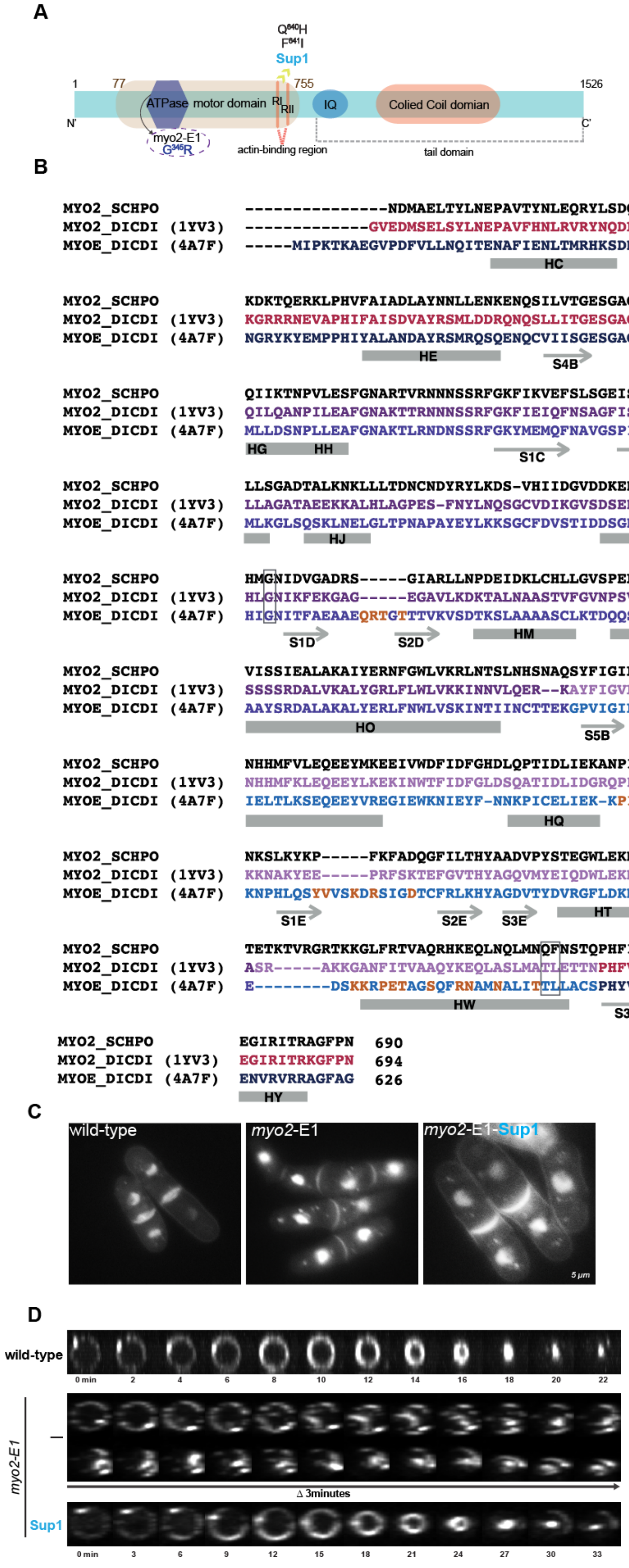
A
Q840H
F841I
Sup1
1
77
755
1526
ATPase motor domain
RI
RII
IQ
Colied Coil domain
N'
C'
myo2-E1
G345R
actin-binding region
tail domain
B
MYO2_SCHPO
MYO2_DICDI (1YV3)
MYOE_DICDI (4A7F)
C
wild-type
myo2-E1
myo2-E1-Sup1
5 μm
D
wild-type
0 min
myo2-E1
Δ 3minutes
Sup1

Figure S1 (related to Figure 1): Schematic representation of myosin II mutants and 3D-projected rings from wild-type, *myo2*-E1 and *myo2*-E1-Sup1.

A. Schematic of type-II myosin (Myo2) domains. The *myo2*-E1 mutation site (G345R), and the intragenic suppressor site (*myo2*-E1-Sup1[Q640H; F641I]), identified via a UV based screen, are highlighted.
B. Multiple sequence alignment of S. *pombe* Myo2p, *Dictyostelium discoideum* Myo2p and MyoEp protein sequences. The *myo2*-E1 (G345R) and the intragenic suppressor *myo2*-E1-Sup1 are marked with boxes around them. The actin-interacting residues are highlighted in orange, while the domain organization is color coded as in Figure S2. The residues missing in the crystal structure are shown in lowercase letters.
C. Cells were grown at 24°C and shifted for 4-6h at 36°C before PFA fixation. DAPI and anillin blue staining used to visualize the nucleus and septum of wild type, *myo2*-E1 and *myo2*-E1-Sup1 intragenic suppressors cells respectively.
D. Time-series of 3D projected end-on view of actomyosin rings from wild-type, *myo2*-E1 and *myo2*-E1-Sup1 cells. Scale bars represent 3 μm.

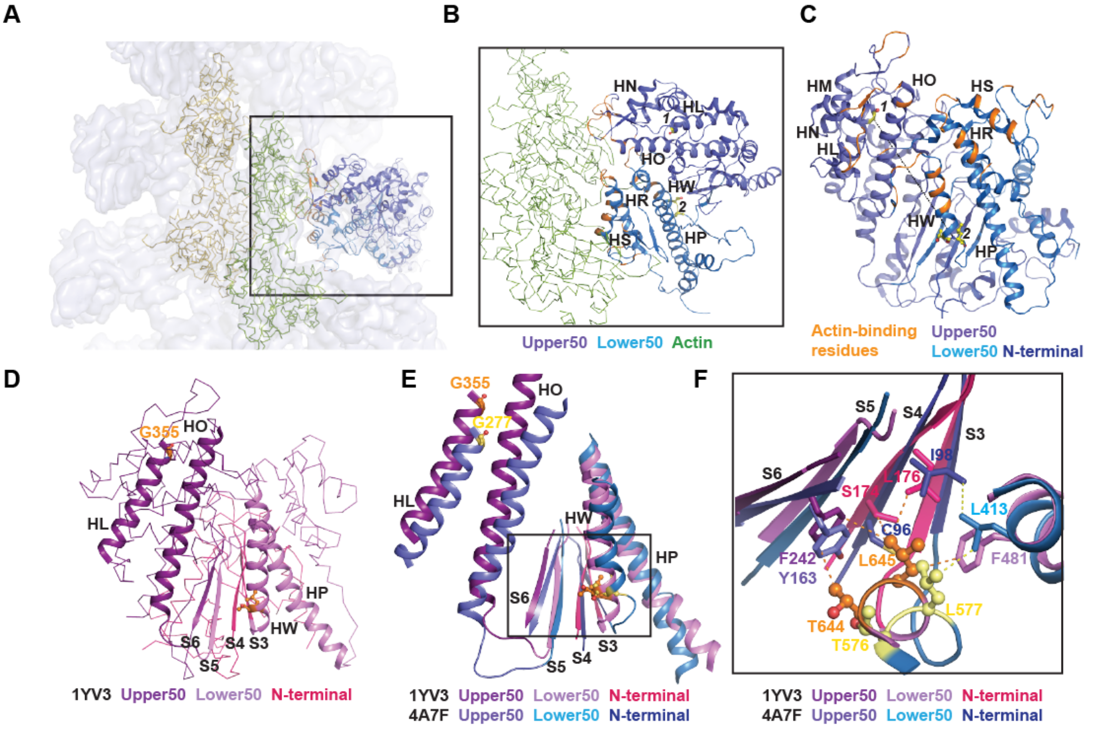

Figure S2 (related to Figure 2 and 3): Analysis of available myosin II and actin structures provides insight into the ring-contraction defect of *myo2*-E1-Sup1.

A. Cryoelectron microscopy reconstruction of a section of actomyosin structure (EMD-1987; PDB ID 4A7F). The subunits in the two protofilaments of actin are shown in yellow and green, while one of the myosin heads is shown in cartoon representation (refer to Supplementary Figure 1B for details of color coding of domains and secondary structure labeling). The residues at the actin-binding interface are highlighted in orange.
B. Zoomed-in view of the actin-myosin interface with the key secondary structure elements labeled. Residues corresponding to G345 (*myo2*-E1) and Q640-F641 (*myo2*-E1-Sup1) are highlighted in stick representation in yellow (labeled as 1 and 2 respectively).
C. The actin-binding interface of myosin involves residues from the Upper 50-K and Lower 50-K sub-domains, and hence relative movement between the two sub-domains contributes to binding efficiency. The distance between *myo2*-E1 and *myo2*-E1-Sup1 is highlighted by the dashed line and corresponds to 36 Å. It is to be noted that the side chains of Q640H and F641I point away from the actin-binding interface.

D. Key secondary structure elements of the myosin Upper 50-K and Lower 50-K sub-domains that undergo conformational changes during the cross-bridge cycle of myosin are highlighted in cartoon representation in a ribbon model of Dictyostelium discoideum myosin II (1YV3). Except for strand S4 of the transducer sheet, the N-terminal domain is not shown for clarity.
E. Key secondary structure elements in (D) are shown. The location of *myo2*-E1 (labeled as G355 and G277 in 1YV3 and 4A7F, respectively) and *myo2*-E1-Sup1 (shown in stick representation on helix HW) are highlighted.
F. Zoomed view of the box in (E) highlights the neighbouring residues of *myo2*-E1-Sup1. The residues T644 and L645 in 1YV3, and T576 and L577 in 4A7F structures correspond to Q640 and F641 of S. pombe Myo2.

A

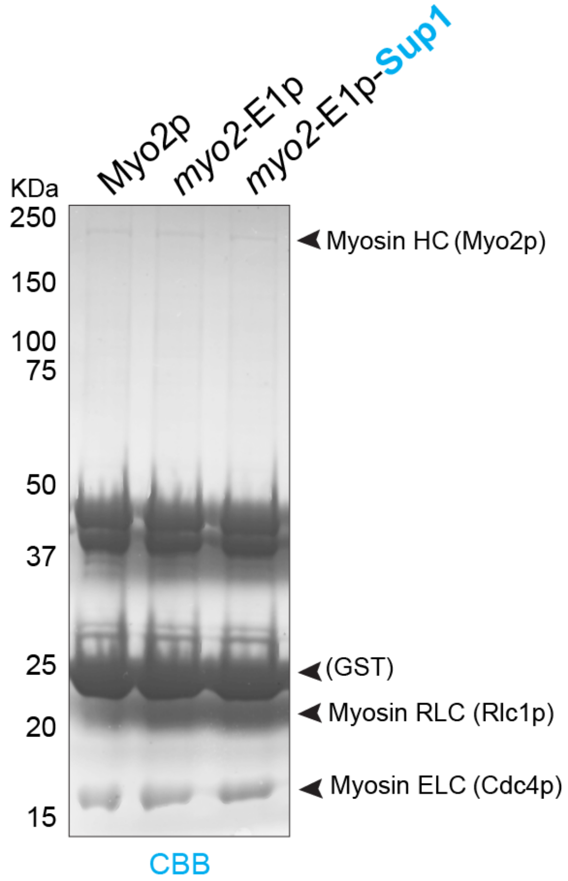

B

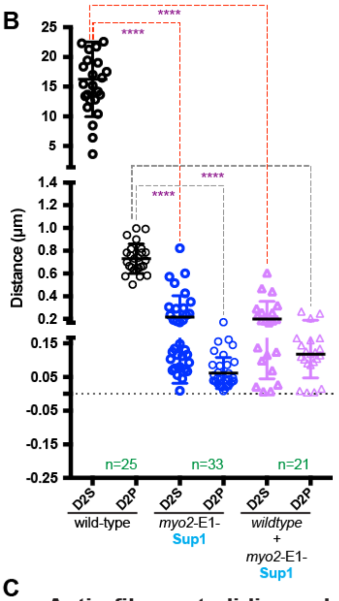

C

Actin filament gliding velocities

Myosin II variants	Gliding velocity (µm/sec) ± SD
**Myo2 (wildtype)**	**0.72±0.13**
***myo2*-E1**	**No filaments attached**
***myo2*-E1-Sup1**	**0.06±0.04**

Figure S3 (related to Figure 4): Myosin purification and gliding assay.

A. SDS-PAGE of purified Type-II myosin complex (Myo2p/Cdc4p/Rlc1p) proteins stained with Coomassie brilliant blue (CBB, SimplyBlue Safestain, Invitrogen). Three lanes show samples from three different myosin complex variants with their light chains. All of them were co-overexpressed using the *nmt41* thiamine repressible promoter for 28-36 hr at 24°C. One step purified myosin complexes (1 mg/ml) were obtained after removal of the GST tag from the light chains.
B. Quantification of the actin filament gliding velocities of Figure 4A. D2S (distance from the initial point of the track to the current point) and D2P (distance from the current point to the preceding point of the track) were plotted separately for the indicated purified myosin variants (Myo2p, myo2-E1p-Sup1, Myo2p+myo2-E1p-Sup1).
C. Actin filament gliding velocities were calculated from different myosin variants (n > 20 filaments per sample).

Supplemental experimental procedures

Yeast genetics and culture methods:

Cells were grown and cultured in Yeast Extract Medium (YES). For the selection of plasmids, cells were grown in Edinburgh Minimal Medium (EMM) with appropriate supplements, as previously described [S1]. ELN (Extremely Low Nitrogen) plates have the same composition as EMM, except that NH_4Cl (50mg/liter) were mainly used for mating and sporulation.

Screen for intragenic suppressors of *myo2*-E1:

Log phase *myo2*-E1 clp1Δ cells plated on YEA agar plates (~2000 cells/plate) were exposed to UV light (9000 μJ/cm^2) for 6 seconds in a UV cross linker (UVP CL-1000 UV cross linker). Plates were stored in the dark at 24°C after UV exposure for 16-20 hours prior to shifting the plates to 36°C for 10-12 days. Positive/rescuing colonies were grown on YEA plates after the 10-12 days of growth at 36°C. Colony PCR was performed on the colonies grown at 36°C using myo2 primers and the PCR products were sequenced to identify the intragenic suppressor of *myo2*-E1.

Fluorescence microscopy:

Cells were grown at 24°C in Yeast Extract Medium (YES) to mid-log phase and shifted to 36°C for 6 hours before fixation. For visualization of DAPI and anillin blue staining, cells were fixed with 4% paraformaldehyde (PFA) and permeabilised with 1% Triton-X-100 at room temperature for 10 minutes. Cells were washed thrice with 1X PBS without detergent and stained with DAPI to visualize DNA and anillin blue to visualize septa and cell wall. Still images were acquired using a wide-field fluorescent microscope (Andor Revolution wide-field imaging system, Andor), equipped with a 100x oil immersion 1.49NA Nikon TIRF Apo objective, an Andor sCMOS ZYLA detector, and Andor iQ software). More than 250 cells were counted per time point. Imaging software ImageJ or Fiji was used to process the images. Where appropriate, statistical significance was determined using Student's t-test (****$P<0.0001$). PRISM 6.0 software (GraphPad) was used for quantification (s.d. = standard deviation).

Live cell imaging:

For time-lapse live cell imaging, mid log phase cells were grown at 24°C and shifted to 36°C for 4-5 hours prior to imaging. Time-lapse movies were acquired for 3-4 hours under a fully controlled 36°C incubation chamber. During imaging, the cells were kept in CellASIC microfluidic yeast plates (Y04C and D size) or in Ibidi microscopic small chambers using a mineral oil suspension method. Time-lapse series were acquired using a spinning disk confocal microscope (Andor Revolution XD imaging system, equipped with a 100x oil immersion 1.45NA Nikon Plan Apo lambda objective, a Confocal Yokogawa CSU-X1 unit, an Andor sCMOS ZYLA detector, and Andor iQ software). When using Cell ASIC plates, Z-stacks comprised of fifteen 0.5 μm

spaced slices were generated for *Rlc1*-3GFP and mCh-atb2 at 1 minute intervals.

Cell ghost preparation and ATP treatment:

Cells were grown at 24°C on minimal medium and rings were isolated as previously described [S2, S3]. Cell ghosts were prepared from wild-type, *myo2*-E1, *myp2*Δ, *myo2*-E1 *myp2*Δ, *myo2*-E1-Sup1 and *myo2*-E1-Sup1 *myp2*Δ cells. *Rlc1*-3GFP was used as a ring marker and images shown are maximum intensity projections of z-stacks. Experiments were done at 24°C. Cell ghosts from the wild-type and mutants were treated with 0.5 mM ATP (t=0) and images were acquired every 30 seconds for 5 minutes. Images were processed using Andor iQ and Fiji imaging software.

Protein Purification:

Three individual strains, wild-type myosin Myo2p (MLP 509), motor defective myosin myo2-E1p (MLP469), and a myosin suppressor allele myo2-E1p-Sup1 (MBY11070), all overexpressing Myo2p from a thiamine controlled expression system under control of the full strength *nmt41* promoter, were co-transformed with GST-tagged light chains from the plasmids pGST-cdc4 and pGST-rlc1. Protein purification, GST tag cleavage, and storage of all myosins, together with their light chains, were performed as previously described [S4]. We used a similar method for the one-step purification of crude myosin. One-step purified myosin protein concentration was determined by Bradford assay with BSA as the standard. Purified myosins were resolved in 4-20% SDS-PAGE gradient gels and stained with Coomassie Brilliant Blue (CBB, SimplyBlue Safe stain, Invitrogen).

In vitro motility assay:

We performed the motility assay using the one step purified myosin and rhodamine-phalloidin stabilised actin filaments as previously described [S4, S5]. One step purified myosin was used at 0.25 µg/µl (total crude protein). Dilution buffer was used to normalise the protein concentration of all myosin variants (myo2-E1p and myo2-E1p-Sup1). Motility of the actin filaments was visualised by spinning disk confocal microscopy (Andor Revolution XD imaging system, equipped with a 100x oil immersion 1.45NA Nikon Plan Apo lambda objective, a Confocal Yokogawa CSU-X1 unit, an Andor sCMOS ZYLA detector, and Andor iQ software) and recorded at one second intervals. Movies were processed using FIJI (ImageJ) and the MTrackJ plug-in [S6]. Individual filament gliding velocities were quantified (n > 20 filaments).

In vitro gliding assay kymographs:

First, filament movement trajectories were visualized through a SUM projection of 20-30 frames of the raw time-lapse images (corresponding to a 20-30 seconds time interval). Next, a linear ROI was manually drawn along the trajectory of the chosen filament. Finally, a kymograph of filament displacement was created with the KymoResliceWide plugin in Fiji (http://imagej.net/KymoResliceWide) using the original time-lapse image and the previously defined ROI with a line thickness of 1.5 µm.

List of strains used in this study

MBY192	*ura4*-D18, *leu1*-32, h-	Lab stock
MBY8841	mCherry-*atb2*::hph; *rlc1*-3GFP::kanMx6; ura4-D18 ade6-210 leu1-32 h+	This study
MBY977	*clp1*::Ura4+, *ura4*-D18 *leu1*-32 *ade6*-21X h+	[S14]
MBY151	*myo2*-E1 *ade6*-21x *ura4*-D18 *leu1*-32 *his3*-d h-	[S15]
MBY2117	*myo2*-E1 *clp1*::*ura4*+ *ade6*-21X *ura4*-D18 *leu1*-32	[S16]
MBY10024	*myo2*-E1[G345R] mCherry-*atb2*::hph; *rlc1*-3*GFP*::*kanMx6*; *ura4* -D18 *ade6*-210 *leu1*-32	This study
MBY8932	*myo2*-E1[G345R]-Sup1[Q640H-F641I] mCherry-atb2::hph; *rlc1-3GFP::kanMx6*; *ura4* -D18 *ade6*-210 *leu1*-32 h-	This study
MBY10075	*myp2*::natMX6 mCherry-*atb2::hph*; *rlc1-3GFP::KanMX6 ade6*-21X	This study
MBY10077	*myp2*::natMX6 *myo2*-E1 mCherry-*atb2*::hph, rlc1-3GFP::KanMX6 ade6-21	This study
MBY10085	*myp2*::natMX6 *myo2*-E1[G345R]-Sup1[Q640H-F641I] mCherry-atb2::hph; Rlc1-3GFP::KanMX6 ade6-21X	This study
MLP 509	leu1-32 ura4::kanR natR:41nmt1prom-*myo2* h-	[S4]
MLP 469	leu1-32 his7-366 ura4-D18 ade6-M216 natR:41nmt1prom-*myo2*-E1 h-	[S4]
MBY11070	leu1-32 his7-366 natR:41nmt1prom-*myo2*-E1-Sup1 (Q640H F641I) h?	This study
MBY11074	pDS472-URA4-Rlc1; pDS473-LEU2-Cdc4 was transformed in to MLP 509 (natR::41nmt1prom-*myo2*) h-	This study
MBY11075	pDS472-URA4-Rlc1; pDS473-LEU2-Cdc4 was transformed in to MLP 469 (natR::41nmt1prom-*myo2*-E1) h-	This study
MBY11076	pDS472-URA4-Rlc1; pDS473-LEU2-Cdc4 was transformed in to MBY11070 (natR::41nmt1prom-*myo2*-E1-Sup1 [Q640H; F641I]) h?	This study

Structure analysis and illustration:

The myosin structures were downloaded from PDB (Protein Data Bank) and EMDB (Electron microscopy database). Structural analysis and illustrations were carried out using PyMOL (Schrodinger). Domain wise structural superpositions were performed for observing relative domain movements. The multiple sequence alignment shown in the Supplementary Figure S1B was obtained using PROMALS3D [S7].

In Myo2-E1p (product of myo2-E1), the bulky side chain of arginine in the place of glycine (G345R) constrains the relative movements between helices HO and HL of the upper 50 kDa sub-domain (Figure S2). This in turn may restrict the interdomain movements between the upper 50 kDa and lower 50 kDa sub-domains, which are essential for actin binding and ATP hydrolysis by Myo2p (Figure S2A-C). The myo2-E1-Sup1 mutations Q640H and F641I are located towards the C-terminal end of the helix HW of the lower 50 kDa sub-domain (Figure S2), far apart from G345 (~36 Å, Figure S2C). These residues are not directly located at the actin-binding interface or near the ATP-binding site. Figure S2A-C highlights the relative position of the mutations and the actin-binding interface of myosin (PDB ID 4A7F; EMD-1987; [S8]) .

Domain-wise structural superposition of various conformations of published myosin crystal structures demonstrates that helices HL (which contains G345), HO in the upper 50 kDa sub-domain, strands 3-6 of the transducer sheet, helix HW, and helix HP (relay helix) undergo key coupled conformational changes (Figure S2D), thus playing a significant role in the allosteric changes during the conformational cycle of myosin. Shown in Figure S2 E and F is the superposition of the key secondary structures mentioned above on the structures of actin-bound myosin IE (PDB ID 4A7F and EMDB ID EMD-1987; [S8]) and myosin II bound to blebbistatin and an ATP analogue (PDB ID 1YV3; [S9]) from Dictyostelium discoideum. The structures were chosen as representatives of the actin-bound and unbound conformations of myosin. Helices HL and HO in the upper 50 kDa sub-domain undergo concerted movement. The conformational movement in the upper 50 kDa sub-domain is coupled to changes in the twist of the transducer beta-sheet, and a kink formation within the relay helix (HP) during the myosin conformational cycle [S10-S12]. Analysis of the crystal structures shows that residues equivalent to Q640 and F641 occupy key positions that could potentially affect these conformational transitions. The equivalent residues corresponding to the F641 side chain (L577/L645 in Figure S2) interact with the residues at the kink of helix HP, and that of Q640 with residues that interact with the transducer sheet (T576/T644 in Figure S2). The kinking movement of the relay helix is crucial in coupling the actin-binding and ATP hydrolysis states of myosin with the conformations of the converter domain and the lever arm. Thus, changes at the position corresponding to Q640 and F641 in myosin might rescue its ability to bind to actin by relieving the constraints imposed by the G345R mutation and promoting a rigor-like state with increased affinity for F-actin, leading to reduced contraction. However, the mutations in Myo2E1-Sup1 appear to have resulted in an increased

affinity for actin due to the increased hydrophobic interactions of the lower 50 kDa sub-domain with the transducer sheet and the relay helix. This increase in affinity to actin thereby led to the defects observed in the contraction of the actomyosin ring.

Well-characterized mutations that affect the coupling between actin-binding and ATP-hydrolysis with the conformations of the converter domain and the lever arm, and thereby with translocation, are the R703C and N93K mutations [S13]. Residues equivalent to R703 and N93 are also implicated in influencing the position of the relay helix in response to the ATP state of myosin. Analysis of R703 equivalent residues in myosin crystal structures show that it communicates to the N-terminal domain (where ATP hydrolysis takes place) through main chain interactions with N93 [S13]. This dictates the position of helix HY, where R703 is located, and subsequently the relay helix and the converter. Similarly, the location of the mutations in myo2-E1-Sup1 mutants reported in this work also communicates to the N-terminal domain through interactions with the strands of the transducer sheet and the relay helix through the hydrophobic patch at its kink. Hence, it is probable that Myo2-E1-Sup1p may function similarly to these R703C and N93K mutants.

Supplemental References:

S1. Moreno, S., Klar, A., and Nurse, P. (1991). Molecular genetic analysis of fission yeast Schizosaccharomyces pombe. Methods Enzymol *194*, 795-823.

S2. Huang, J., Mishra, M., Palani, S., Chew, T.G., and Balasubramanian, M.K. (2016). Isolation of Cytokinetic Actomyosin Rings from Saccharomyces cerevisiae and Schizosaccharomyces pombe. Methods Mol Biol *1369*, 125-136.

S3. Mishra, M., Kashiwazaki, J., Takagi, T., Srinivasan, R., Huang, Y., Balasubramanian, M.K., and Mabuchi, I. (2013). In vitro contraction of cytokinetic ring depends on myosin II but not on actin dynamics. Nat Cell Biol *15*, 853-859.

S4. Lord, M., and Pollard, T.D. (2004). UCS protein Rng3p activates actin filament gliding by fission yeast myosin-II. J Cell Biol *167*, 315-325.

S5. Tang, Q., Pollard, L.W., and Lord, M. (2016). Measurements of Myosin-II Motor Activity During Cytokinesis in Fission Yeast. Methods Mol Biol *1369*, 137-150.

S6. Meijering, E., Dzyubachyk, O., and Smal, I. (2012). Methods for cell and particle tracking. Methods Enzymol *504*, 183-200.

S7. Pei, J., Kim, B.H., and Grishin, N.V. (2008). PROMALS3D: a tool for multiple protein sequence and structure alignments. Nucleic Acids Res *36*, 2295-2300.

S8. Behrmann, E., Muller, M., Penczek, P.A., Mannherz, H.G., Manstein, D.J., and Raunser, S. (2012). Structure of the rigor actin-tropomyosin-myosin complex. Cell *150*, 327-338.

S9. Allingham, J.S., Smith, R., and Rayment, I. (2005). The structural basis of blebbistatin inhibition and specificity for myosin II. Nat Struct Mol Biol *12*, 378-379.

S10. Yang, Y., Gourinath, S., Kovacs, M., Nyitray, L., Reutzel, R., Himmel, D.M., O'Neall-Hennessey, E., Reshetnikova, L., Szent-Gyorgyi, A.G., Brown, J.H., et al. (2007). Rigor-like structures from muscle myosins reveal key mechanical elements in the transduction pathways of this allosteric motor. Structure *15*, 553-564.

S11. Sweeney, H.L., and Houdusse, A. (2010). Structural and functional insights into the Myosin motor mechanism. Annu Rev Biophys *39*, 539-557.

S12. Preller, M., and Manstein, D.J. (2013). Myosin structure, allostery, and mechano-chemistry. Structure *21*, 1911-1922.

S13. Kim, K.Y., Kovacs, M., Kawamoto, S., Sellers, J.R., and Adelstein, R.S. (2005). Disease-associated mutations and alternative splicing alter the enzymatic and motile activity of nonmuscle myosins II-B and II-C. J Biol Chem *280*, 22769-22775.

S14. Trautmann, S., Wolfe, B.A., Jorgensen, P., Tyers, M., Gould, K.L., and McCollum, D. (2001). Fission yeast Clp1p phosphatase regulates G2/M transition and coordination of cytokinesis with cell cycle progression. Curr Biol *11*, 931-940.

S15. Balasubramanian, M.K., McCollum, D., Chang, L., Wong, K.C., Naqvi, N.I., He, X., Sazer, S., and Gould, K.L. (1998). Isolation and

characterization of new fission yeast cytokinesis mutants. Genetics *149*, 1265-1275.

S16. Mishra, M., Karagiannis, J., Trautmann, S., Wang, H., McCollum, D., and Balasubramanian, M.K. (2004). The Clp1p/Flp1p phosphatase ensures completion of cytokinesis in response to minor perturbation of the cell division machinery in Schizosaccharomyces pombe. J Cell Sci *117*, 3897-3910.
