# Supplementary figures and images for: Motor Activity Dependent and Independent Functions of Myosin II Contribute to Actomyosin Ring Assembly and Contraction in Schizosaccharomyces pombe
Source: Curr Biol. 2017 Mar 6;27(5):751–7. doi: 10.1016/j.cub.2017.01.028 (PMC5344676; doi:10.1016/j.cub.2017.01.028)

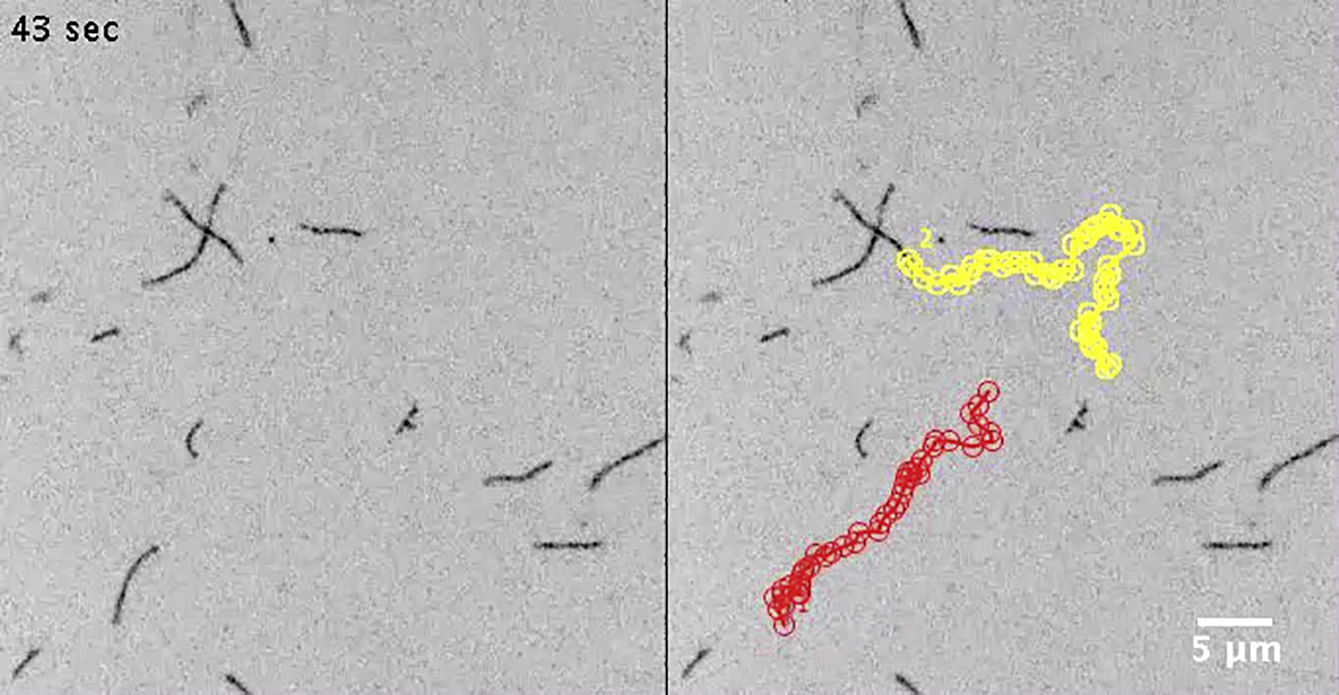

Supplement: Movie S1. Filament-Gliding Assay of One-Step-Purified Crude Wild-Type Myosin Myo2p/Cdc4p/Rlc1p Complex, Related to Figure 4 — Time-lapse movie of polymerized rhodamine-phalloidin labelled rabbit-skeletal muscle actin filaments in the presence of crude wild-type Myo2p and its associated light chains. Gliding assay was performed as described by Tang et al. (2016) [S5]. [file mmc2.jpg]

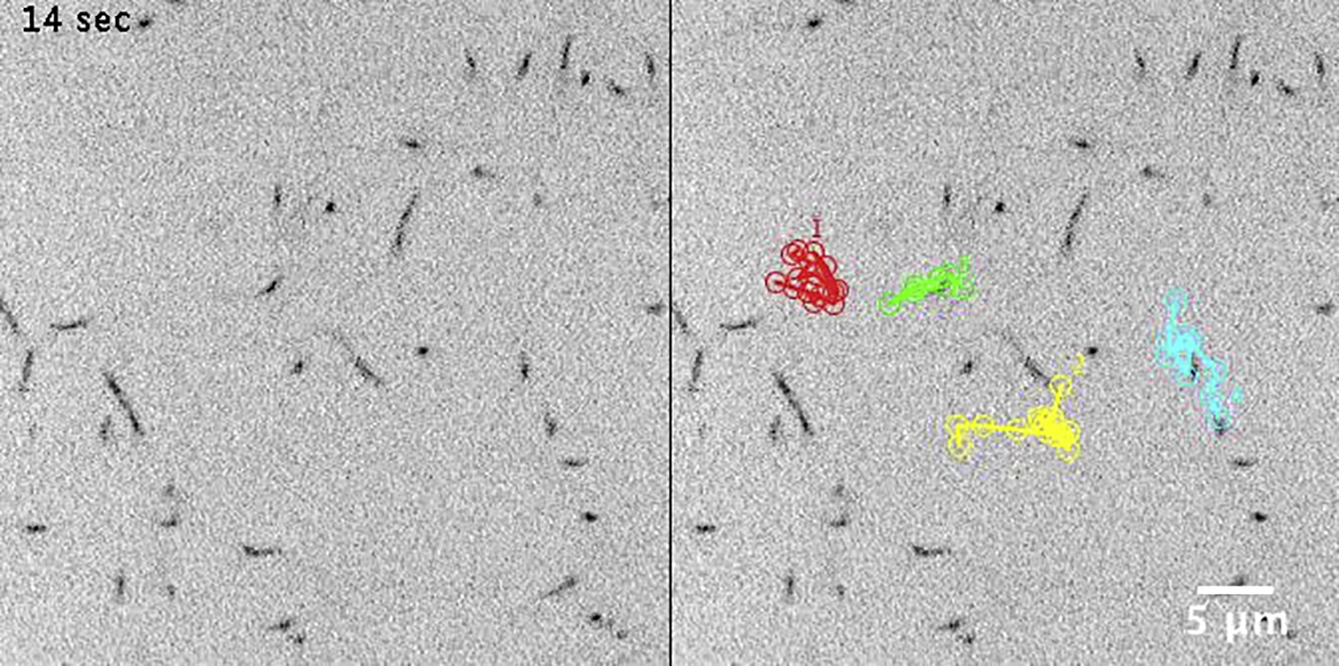

Supplement: Movie S2. Product of Motor-Defective Allele of myo2, Myo2-E1p, Did Not Bind Actin Filaments, Related to Figure 4 — The product of myo2-E1 and its associated light chains (Myo2-E1p/Cdc4p/Rlc1p) was purified at 24°C and the gliding assay was performed as described by Tang et al. (2016) [S5]. Rhodamine-phalloidin labelled rabbit-skeletal muscle actin filaments did not bind to Myo2-E1p. [file mmc3.jpg]

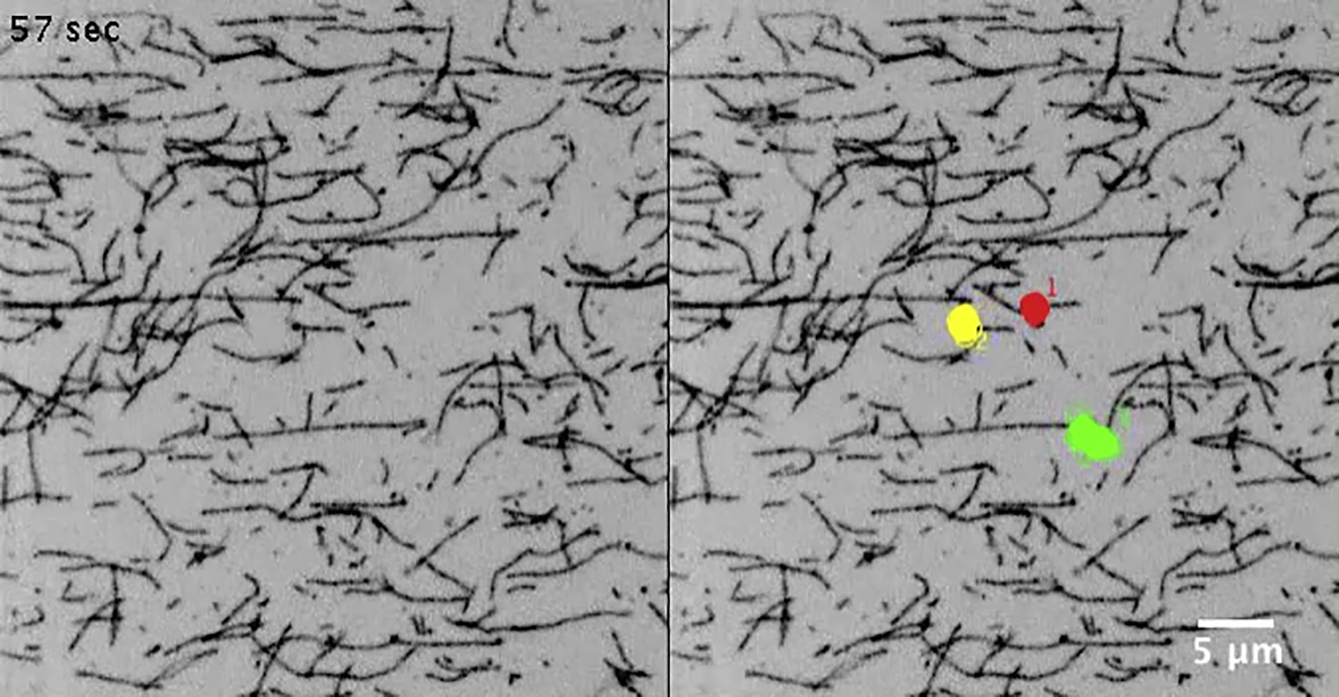

Supplement: Movie S3. Myo2-E1p-Sup1 Bound, but Did Not Move, Actin Filaments, Related to Figure 4 — The product of myo2-E1-Sup1 and its associated light chains (Myo2-E1p/Cdc4p/Rlc1p) was purified at 24°C and the gliding assay was performed as described by Tang et al. (2016) [S5]. Rhodamine-phalloidin labelled rabbit-skeletal muscle actin filaments was found to bind tightly to Myo2-E1p-Sup1 and its associated light chains, but these filaments were not translocated by Myo2-E1-Sup1p. [file mmc4.jpg]

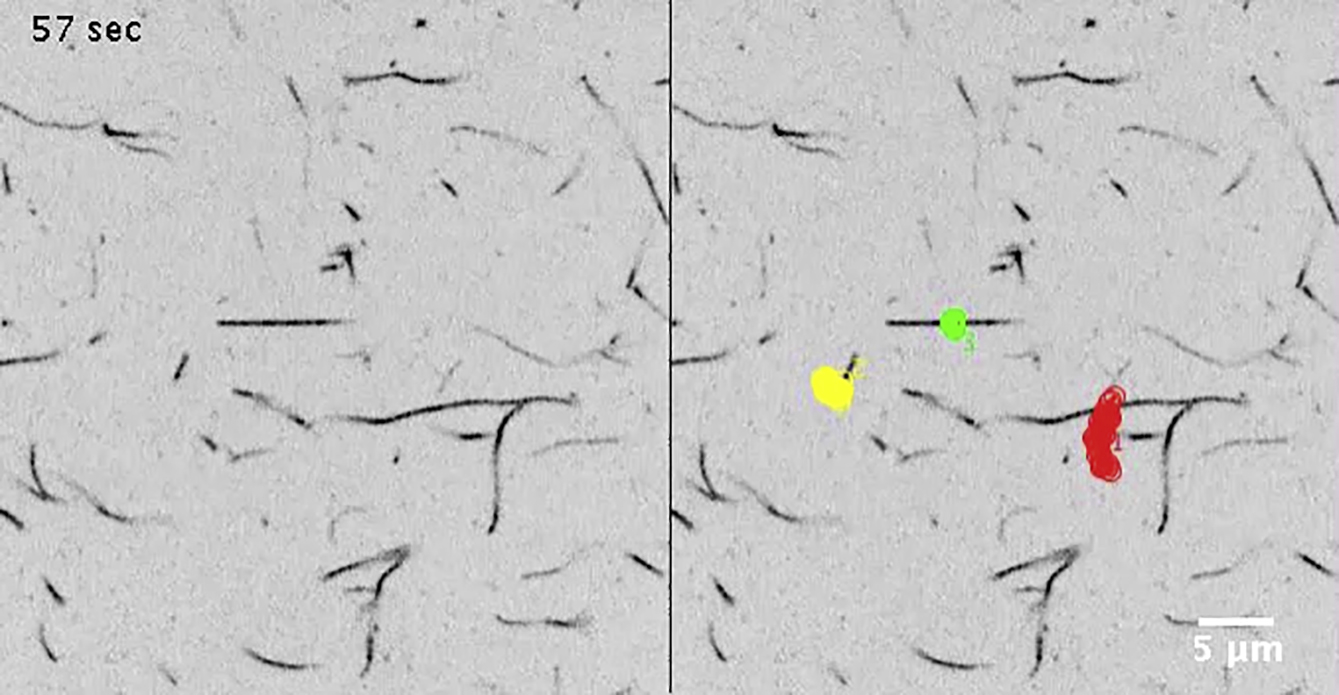

Supplement: Movie S4. A Mixture of Myo2p and Myo2-E1p-Sup1p Did Not Support Actin Motility, Related to Figure 4 — Pre-mixed 1:1 myosin complex (Myo2p::Myo2-E1p-Sup1) was found to bind tightly to rhodamine-phalloidin labelled actin filaments but fail to move the filaments. [file mmc5.jpg]
