# Supplementary material for: Motor Activity Dependent and Independent Functions of Myosin II Contribute to Actomyosin Ring Assembly and Contraction in Schizosaccharomyces pombe
Source: Curr Biol. 2017 Mar 6;27(5):751–7. doi: 10.1016/j.cub.2017.01.028 (PMC5344676; doi:10.1016/j.cub.2017.01.028)
Supplement: Document S2. Article plus Supplemental Information [file mmc6.pdf]

Report

Current Biology

Motor Activity Dependent and Independent Functions of Myosin II Contribute to Actomyosin Ring Assembly and Contraction in *Schizosaccharomyces pombe*

Highlights

- In many eukaryotes, cytokinesis requires an actomyosin-based contractile ring
- The role of motor activity of myosin II in cytokinesis is a topic of active debate
- We isolate a new allele of *S. pombe* Myo2, an essential myosin heavy chain
- We show motor activity-dependent and -independent roles for Myo2

Authors

Saravanan Palani, Ting Gang Chew, Srinivasan Ramanujam, ..., Mithilesh Mishra, Pananghat Gayathri, Mohan K. Balasubramanian

Correspondence

s.palani@warwick.ac.uk (S.P.), m.k.balasubramanian@warwick.ac.uk (M.K.B.)

In Brief

Cytokinesis in many eukaryotes requires an actomyosin-based contractile ring. The role of the motor protein Myosin II in cytokinesis is actively debated. Palani et al. identify a new motor activity-defective allele of fission yeast myosin II and report that the motor activity is dispensable for ring assembly but is essential for ring contraction.

CrossMark

Palani et al., 2017, Current Biology *27*, 751–757
March 6, 2017 © 2017 The Author(s). Published by Elsevier Ltd.
http://dx.doi.org/10.1016/j.cub.2017.01.028

CellPress

Current Biology
Report

Motor Activity Dependent and Independent Functions of Myosin II Contribute to Actomyosin Ring Assembly and Contraction in *Schizosaccharomyces pombe*

Saravanan Palani,[1,*] Ting Gang Chew,[1] Srinivasan Ramanujam,[2] Anton Kamnev,[1] Shrikant Harne,[5] Bernardo Chapa-y-Lazo,[1] Rebecca Hogg,[1] Mayalagu Sevugan,[3] Mithilesh Mishra,[3,4] Pananghat Gayathri,[5] and Mohan K. Balasubramanian[1,6,*]
[1]Division of Biomedical Sciences, Warwick Medical School, University of Warwick, Coventry CV4 7AL, UK
[2]School of Biological Sciences, National Institute of Science Education and Research (NISER), Odisha 752050, India
[3]Temasek Life Sciences Laboratory, 1. Research Link, National University of Singapore, Singapore 117604, Singapore
[4]Department of Biological Sciences, Tata Institute of Fundamental Research (TIFR), Mumbai, Maharashtra 400005, India
[5]Biology Division, Indian Institute of Science Education and Research (IISER), Pune, Maharashtra 411008, India
[6]Lead Contact
*Correspondence: s.palani@warwick.ac.uk (S.P.), m.k.balasubramanian@warwick.ac.uk (M.K.B.)
http://dx.doi.org/10.1016/j.cub.2017.01.028

SUMMARY

Cytokinesis depends on a contractile actomyosin ring in many eukaryotes [1–3]. Myosin II is a key component of the actomyosin ring, although whether it functions as a motor or as an actin cross-linker to exert its essential role is disputed [1, 4, 5]. In *Schizosaccharomyces pombe*, the *myo2*-E1 mutation affects the upper 50 kDa sub-domain of the myosin II heavy chain, and cells carrying this lethal mutation are defective in actomyosin ring assembly at the non-permissive temperature [6, 7]. *myo2*-E1 also affects actomyosin ring contraction when rings isolated from permissive temperature-grown cells are incubated with ATP [8]. Here we report isolation of a compensatory suppressor mutation in the lower 50 kDa sub-domain (*myo2*-E1-Sup1) that reverses the inability of *myo2*-E1 to form colonies at the restrictive temperature. *myo2*-E1-Sup1 is capable of assembling normal actomyosin rings, although rings isolated from *myo2*-E1-Sup1 are defective in ATP-dependent contraction in vitro. Furthermore, the product of *myo2*-E1-Sup1 does not translocate actin filaments in motility assays in vitro. Superimposition of *myo2*-E1 and *myo2*-E1-Sup1 on available rigor and blebbistatin-bound myosin II structures suggests that *myo2*-E1-Sup1 may represent a novel actin translocation-defective allele. Actomyosin ring contraction and viability of *myo2*-E1-Sup1 cells depend on the late cytokinetic *S. pombe* myosin II isoform, Myp2p, a non-essential protein that is normally dispensable for actomyosin ring assembly and contraction. Our work reveals that Myo2p may function in two different and essential modes during cytokinesis: a motor activity-independent form that can promote actomyosin ring assembly and a motor activity-dependent form that supports ring contraction.

RESULTS AND DISCUSSION

The product of the *myo2*-E1 allele is predicted to harbor a substitution of glycine at position 345 with arginine (Figures S1A and S1B). Cells carrying this mutant allele are capable of colony formation at 24°C but are severely compromised for colony formation at 36°C (Figure 1A) due to defective actomyosin ring assembly [6, 7, 9, 10]. The *myo2*-E1 mutation resides between α-helix HL and β sheet S1D, which is part of the upper 50 kDa sub-domain in the head of Myo2p (Figure S1B). Previous work has shown that *Myo2*-E1p (product of myo2-E1) does not bind or move actin filaments and has a very low ATPase activity in vitro [10, 11]. The presence of a bulky arginine side chain between helices HL and HO in the upper 50 kDa sub-domain of this mutant might introduce constraints to the conformational changes in the Myo2p head domain during the actomyosin cycle, resulting in the observed phenotypes. To further understand the role of Myo2p in cytokinesis, we isolated genetic suppressors that restored the ability of *myo2*-E1 cells to form colonies at 36°C (Figure 1A). One suppressor, *myo2*-E1-Sup1, is described in this study. Genetic crosses between *myo2*-E1-Sup1 and wild-type cells only produced progeny that were able to form colonies at 36°C, suggesting that the suppressor mutation was intragenic or very tightly linked to *myo2*. Nucleotide sequence determination revealed that *myo2*-E1-Sup1 contained the original G345R mutation and also had additional mutations (Q640H and F641I) (Figures S1A and S1B). Furthermore, no sequence alterations were found in the neighboring *rgf3* gene (data not shown), which has also been implicated in cytokinesis [12, 13]. Therefore, we concluded that the sequence alteration Q640H F641I was responsible for the suppression of *myo2*-E1. Interestingly, Q640H and F641I are located in the HW region of the Myo2p head (within the lower 50 KDa sub-domain), which is at a significant distance (∼36 Å) from HL and S1D, the region where the original mutation resides, suggesting

This is an open access article under the CC BY license (http://creativecommons.org/licenses/by/4.0/).

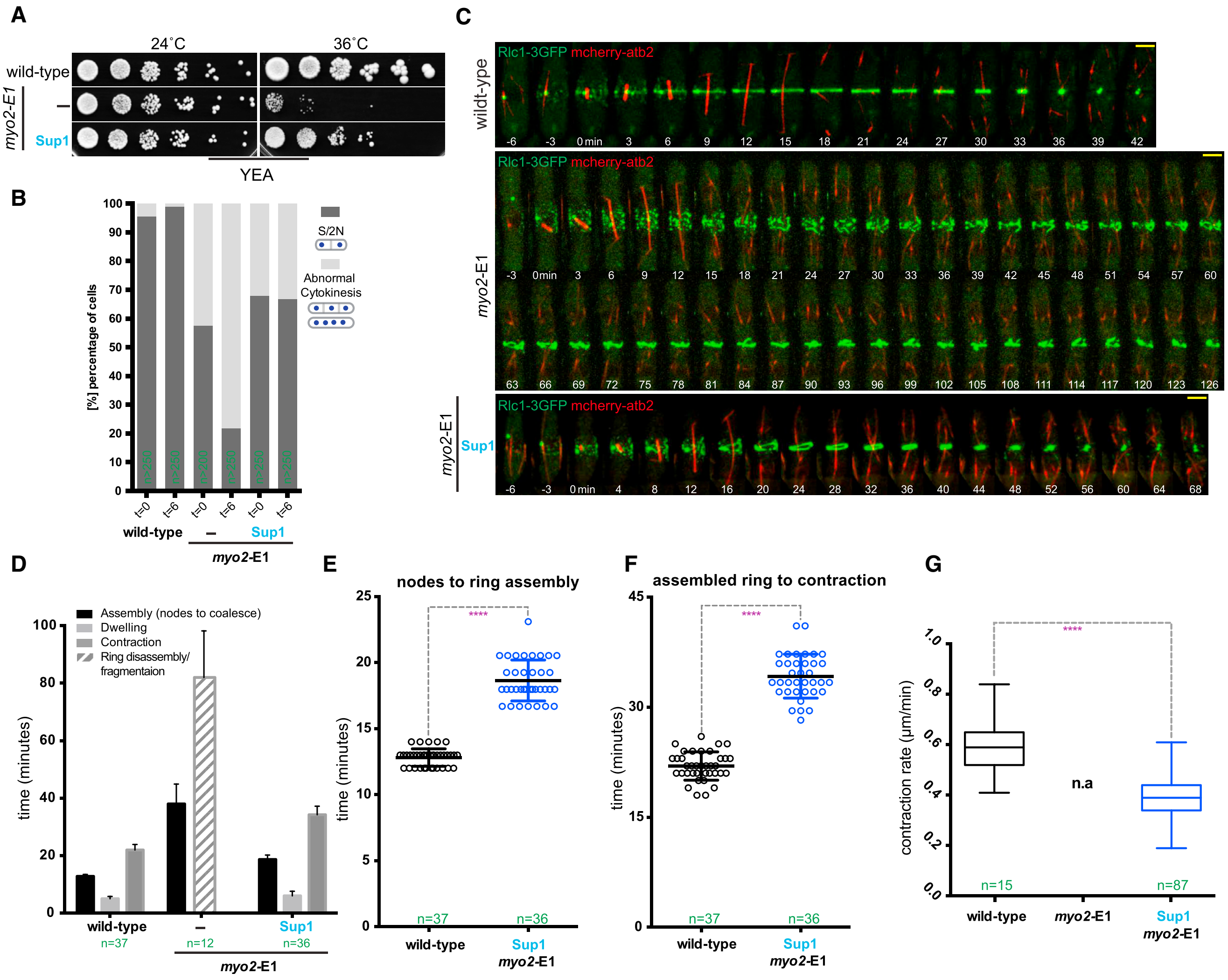

Figure 1. *myo2*-E1-Sup1 Restores Actomyosin Ring Assembly and Partial Ring Contraction

(A) Serial dilutions (10-fold) of wild-type, *myo2*-E1, and the intragenic suppressor *myo2*-E1-Sup1 were spotted onto yeast extract agar (YEA) plates and grown for 3 days at 24°C and 36°C.

(B) Quantification of DAPI and anillin blue staining used to visualize the nucleus and septum of wild-type, *myo2*-E1, and *myo2*-E1-Sup1 cells, respectively. Phenotypes of the mutants were categorized into two types: septa with two nuclei (S/2N) and cells with abnormal cytokinesis, revealed by the presence of multiple septa and nuclei (MS/>2N).

(C) Time-lapse series of wild-type, *myo2*-E1, and *myo2*-E1-Sup1 cells expressing 3GFP-tagged myosin regulatory light chain (Rlc1-3GFP) as a contractile ring marker and mCherry-tagged tubulin (mCherry-atb2) as a cell-cycle stage marker. Cells were grown at 24°C and shifted to 36°C for 3–4 hr before imaging at 36°C (t = 0 indicates the time before Rlc1-3GFP nodes localize to the cell middle). Images shown are maximum-intensity projections of z stacks. Scale bars represent 3 μm.

(D) Timing of contractile ring assembly, maturation/dwelling, and contraction. Quantification of (C) is shown. Error bars represent SD.

(E) Timing of actomyosin ring assembly from nodes. Quantification of (C) is shown (asterisks indicate the statistical significance of the difference between the two genotypes). Statistical significance was calculated by Student's t test (****$p < 0.0001$). Error bars represent SD.

(F) Timing of actomyosin ring contraction. Quantification of (C) is shown. Statistical significance was calculated by Student's t test (****$p < 0.0001$). Error bars represent SD.

(G) Constriction rate determined from a graph of ring circumference versus time. Statistical significance was calculated by Student's t test (****$p < 0.0001$). Error bars represent SD.

See also Figure S1.

potential allosteric mechanisms, rather than a simple reversal of original mutation, may operate in the suppression.

Following a 6 hr shift to 36°C, nearly 80% of *myo2*-E1 cells became multinucleate and had either improper septa with a wavy and patchy appearance or did not have a septum (Figure 1B). By contrast, only ~35% of *myo2*-E1-Sup1 cells contained such defects, while those defects were rarely seen in wild-type cells (Figures 1B and S1C). Since the ingressing actomyosin ring guides division septum assembly, we investigated the dynamics of the actomyosin ring component Rlc1p-3GFP

in wild-type, *myo2*-E1, and *myo2*-E1-Sup1 strains; mCherry-tubulin served as a cell-cycle marker in these experiments. In wild-type cells, actomyosin rings were assembled in metaphase/anaphase A in ~12.8 ± 0.6 min and contracted following spindle breakdown in ~22 ± 1.9 min, with an intervening dwell phase of 5 ± 0.8 min during which the actomyosin ring was stably maintained (Figures 1C–1E and S1D). As expected, all aspects of cytokinesis were slower in myo2-E1 mutants compared to wild-type cells: improper ring assembly took ~38 ± 6.9 min and improper contraction/disassembly lasted ~82 ± 16.2 min at 36°C (Figures 1C and S1D). Imaging *myo2*-E1-Sup1 cells revealed that they assembled actomyosin rings of normal appearance (Figure 1C, time point 24 min, ending on views in Figure S1D), with a significantly accelerated kinetics for both ring assembly (~18.6 ± 1.5 min) and contraction (~34.2 ± 3 min) compared to the original *myo2*-E1 mutant. Nevertheless, both steps were marginally slower in *myo2*-E1-Sup1 compared to wild-type cells (Figures 1C–1E and S1D). Whereas actomyosin rings in wild-type cells contracted at ~0.6 ± 0.1 μm/min, contraction rate in *myo2*-E1-Sup1 cells was ~0.4 ± 0.08 μm/min at 36°C. These experiments established that *myo2*-E1-Sup1 assembled contractile rings of normal appearance, although both ring assembly and ring contraction took ~1.5 times longer compared to wild-type cells.

Two type II myosin heavy chains participate in cytokinesis in *S. pombe* [14–17]. We therefore investigated the possibility that Myp2p, which is normally non-essential for ring assembly, assisted in actomyosin ring assembly and contraction in the *myo2*-E1-Sup1 strain through a potential ectopic upregulation. Toward this goal, we generated a double mutant of the genotype *myo2*-E1-Sup1 myp2Δ. Although this strain was viable at 24°C, surprisingly, it was inviable at 36°C (Figure 2A). Time-lapse microscopy was performed on wild-type, *myo2*-E1 *myp2*Δ, *myo2*-E1-Sup1 *myp2*Δ, and *myp2*Δ strains to investigate aspects of actomyosin ring function. The time taken for ring assembly and contraction and the ring contraction rate were comparable in wild-type and *myp2*Δ cells (Figures 2B–2F), clarifying that Myp2p is not important for either ring assembly or contraction at 36°C when Myo2p is fully functional. *myo2*-E1 *myp2*Δ assembled abnormal actomyosin rings that underwent abnormal disassembly (Figures 2B and 2C). *myo2*-E1-Sup1 *myp2*Δ assembled actomyosin rings of normal appearance, and the assembly of these rings took ~6 min more than wild-type and *myp2*Δ cells (Figures 2B–2D). Ring contraction was dramatically affected in *myo2*-E1-Sup1 *myp2*Δ (Figures 2B, 2C, 2E, and 2F). Contraction and disassembly took more than twice the amount of time compared to wild-type cells, while the ring contraction rate was less than half of that observed in wild-type cells (Figures 2E and 2F). Furthermore, contraction was frequently asymmetric and led to rings disassembling abnormally and often to the fragmentation of the ring into two or more clusters (Figures 2B, time points 48–72 min, and 2C). Since *myo2*-E1-Sup1 *myp2*Δ and *myo2*-E1-Sup1 were capable of actomyosin ring assembly but showed appreciable defects in ring contraction, we conclude Myo2p activity is essential for ring assembly and contraction, whereas Myp2p plays an ancillary role in promoting inefficient contraction when Myo2p motor activity is compromised at 36°C (compare ring contraction times and rates between *myo2*-E1-Sup1 and *myo2*-E1-Sup1 *myp2*Δ in Figures 2E and 2F).

Analysis of three-dimensional structures of rigor myosin (actin bound: 4A7F) and blebbistatin-bound myosin (actin unbound: 1YV3) suggested that the amino acid substitutions in *myo2*-E1-Sup1 may result in increased binding affinity toward F-actin (Figure S2; see the Supplemental Experimental Procedures for a detailed description of the structural analysis). This in turn may lead to defective actomyosin ring contraction due to *myo2*-E1-Sup1 being tightly bound to actin, leading to an actin filament translocation defect.

We have already developed methods to isolate ATP-dependent contraction-competent actomyosin rings [8, 18]. We therefore used this system to test if isolated actomyosin rings in cell ghosts from *myo2*-E1-Sup1 were capable of ATP-dependent contraction. Actomyosin rings were isolated from wild-type, *myo2*-E1, *myp2*Δ, *myo2*-E1 *myp2*Δ, *myo2*-E1-Sup1, and *myo2*-E1-Sup1 *myp2*Δ cells grown at the permissive temperature of 24°C. Actomyosin rings isolated from wild-type and myp2Δ cells underwent normal and rapid contraction upon ATP addition (Figures 3A and 3B). As previously reported [8], upon the addition of 0.5 mM ATP, actomyosin rings isolated from *myo2*-E1 and *myo2*-E1 myp2Δ either contracted slowly or underwent fragmentation (Figures 3A and 3B). Interestingly, despite the moderate delay in ring assembly timing, actomyosin rings of normal appearance assembled in *myo2*-E1-Sup1 and *myo2*-E1-Sup1 *myp2*Δ at the restrictive temperature. However, rings isolated from these strains did not contract normally, even at the permissive temperature for *myo2*-E1 (24°C). Instead, rings from these strains remained stable and broke into large fragments. These experiments established that, consistent with in vivo results, rings isolated from *myo2*-E1-Sup1 and *myo2*-E1-Sup1 *myp2*Δ cells are defective in ATP-dependent contraction in vitro. These results were consistent with the idea that the product of *myo2*-E1-Sup1 is defective in its motor activity and actin filament translocation, but not in actin filament binding, which in turn may explain the ability of *myo2*-E1-Sup1 to support actomyosin ring assembly, but not contraction. However, it was possible that the actin translocation defect in *myo2*-E1-Sup1 was due to allosteric effects on other unidentified components of the actomyosin ring that affect ring contraction, rather than a direct effect of *myo2*-E1-Sup1 on actin filament translocation.

To distinguish between these possibilities, we purified the products of *myo2*$^{+}$, *myo2*-E1, and *myo2*-E1-Sup1 using an expression system developed by Lord and Pollard [11]. *Myo2*-E1-Sup1p was more difficult to purify (potentially due to its tight binding to actin) and was eventually isolated from Latrunculin A-treated cells (Figure S3A). We then performed actin motility assays as described in Lord and Pollard [11]. In brief, Myo2p and the mutant versions were immobilized on nitrocellulose-coated coverslips, overlaid with rhodamine-phalloidin-stabilized rabbit actin filaments, and incubated with ATP (Figures 4A, 4B, S3B, and S3C; Movies S1, S2, S3, and S4). We found that wild-type Myo2p was able to bind and translocate actin filaments at ~0.72 ± 0.13 μm/s when incubated with ATP. Also, as previously reported [11], Myo2-E1p did not attach to actin filaments (Movie S2). Interestingly, unlike the product of *myo2*-E1, the product of *myo2*-E1-Sup1 bound actin tightly, since these filaments were either severely affected for motility or were non-motile (gliding velocity was

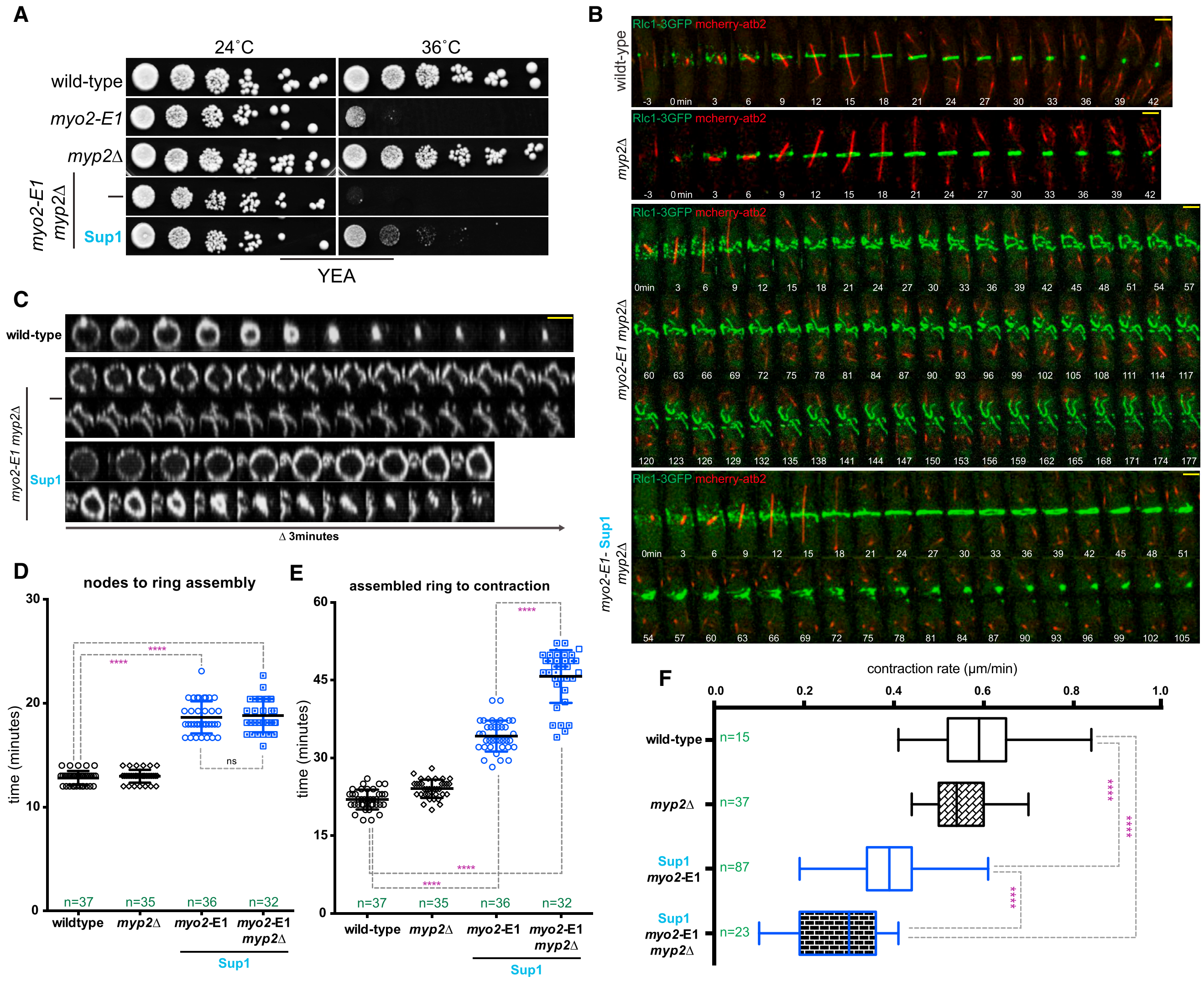

Figure 2. *myo2*-E1-Sup1 Fails in Actomyosin Ring Contraction in the Absence of the Non-essential Myosin Heavy Chain Myp2p

(A) Serial dilutions (10-fold) of wild-type, *myo2*-E1, *myp2*Δ, *myo2*-E1 *myp2*Δ, and *myo2*-E1-Sup1 *myp2*Δ were spotted onto YEA plates and grown for 3 days at 24°C and 36°C.

(B) Time-lapse series of wild-type, *myp2*Δ, *myo2*-E1 *myp2*Δ, and *myo2*-E1-Sup1 *myp2*Δ cells expressing 3GFP-tagged myosin regulatory light chain (Rlc1-3GFP) as a contractile ring marker and mCherry-tagged tubulin (atb2-mCherry) as a cell-cycle stage marker. Cells were grown at 24°C and shifted to 36°C for 3–4 hr before imaging at 36°C (t = 0 indicates the time before Rlc1-3GFP nodes localize to the cell middle). Images shown are maximum-intensity projections of z stacks. Scale bars represent 3 µm.

(C) Kymographs of a 3D-projected ring from wild-type, *myo2*-E1 *myp2*Δ, and *myo2*-E1-Sup1 *myp2*Δ cells. Scale bars represent 3 µm.

(D) Timing of actomyosin ring assembly from nodes. Quantification of (B) is shown. Asterisks indicate the statistical significance of the difference between the different genotypes compared to the wild-type. Statistical significance was calculated by Student's t test (***p < 0.0001). Error bars represent SD.

(E) Timing of actomyosin ring contraction. Quantification of Figure 1C and (B) is shown. Statistical significance was calculated by Student's t test (****p < 0.0001). Error bars represent SD.

(F) Constriction rate determined from a graph of ring circumference versus time. Contraction rates of Figure 1C and (B) are shown. Statistical significance was calculated by Student's t test (****p < 0.0001). Error bars represent SD.

See also Figure S2.

~0.06 ± 0.04 µm/s). Myo2-E1-Sup1p also had a dominant effect when mixed with wild-type Myo2p. The mixture bound to actin filaments but these filaments were non-motile. The fact that Myo2-E1-Sup1p did not support motility, despite binding actin filaments and its dominant-negative effect on motility over wild-type Myo2p, suggests that Myo2-E1-Sup1p is most likely a novel rigor mutant of Myo2p.

Our work reported in this study establishes that the type II myosin, Myo2p, plays two distinct and essential roles. Since cells harboring the novel rigor mutant allele *myo2*-E1-Sup1 assemble normal actomyosin rings, despite the defective contraction in vitro and in vivo, it is possible that actomyosin ring assembly depends on the ability of Myo2p to cross-link actin filaments. Actomyosin ring assembly in *myo2*-E1-Sup1 cells is

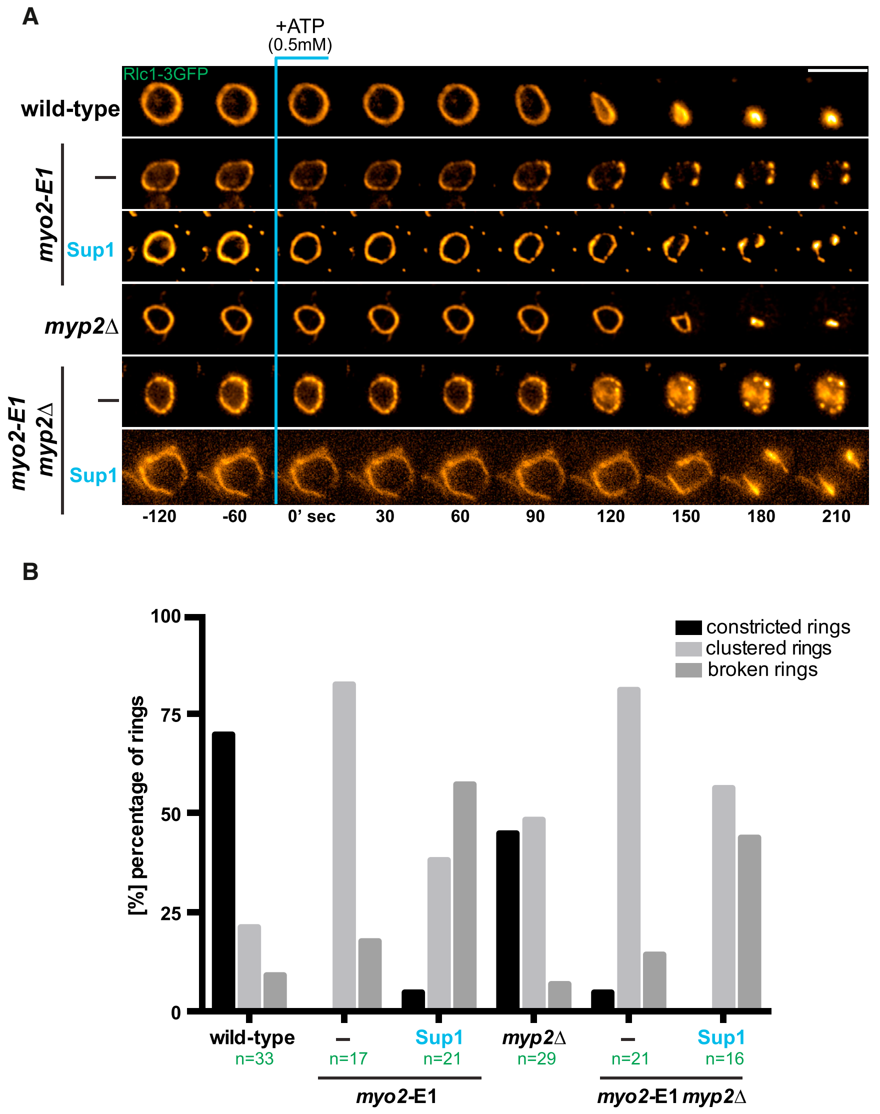

Figure 3. Isolated Actomyosin Rings of *myo2*-E1-Sup1 Do Not Undergo ATP-Dependent Contraction

(A) Cell ghosts were prepared from wild-type, *myo2*-E1, *myp2Δ*, *myo2*-E1 *myp2Δ*, *myo2*-E1-Sup1, and *myo2*-E1-Sup1 *myp2*Δ grown at 24°C. Ring contraction experiments were performed at 24°C and contraction was activated by the addition of 0.5 mM ATP. Images shown are maximum-intensity projections of z stacks. Scale bars represent 5 μm.

(B) Graph showing percentage of contracted, clustered, and broken rings. Quantification of (A) is shown.

See also Figure S2.

slower than in wild-type cells (possibly due to cross-linking and tighter binding of Myo2-E1-Sup1p with actin), suggesting that myosin II motor activity may also play a role in actomyosin ring assembly, as previously proposed [19, 20]. It is possible that clustering of cytokinetic precursor nodes can occur through tension generated by myosin II-dependent cross-linking of actin filaments. This view is consistent with aspects of the work of Ma and colleagues who have proposed that actin translocation activity of myosin II is not essential for cytokinesis [4]. Inconsistent with the work of Ma and colleagues, however, are our findings that actomyosin rings in *myo2*-E1-Sup1 cells do not contract normally, that actomyosin rings isolated from those cells fail to undergo ATP-dependent contraction, and that one-step-purified Myo2-E1-Sup1p does not support ATP-dependent actin filament motility in vitro. These observations suggest that myosin II motor activity is essential for actomyosin ring contraction.

Thus, through the analysis of novel myosin II mutant alleles, we have been able to discriminate between myosin II motor activity-dependent and -independent steps in cytokinesis. Published work in *S. cerevisiae* and mammalian cells [4, 5, 21] has questioned the role of myosin II motor activity in cytokinesis. It is likely that in some cell types, tension generated by actin filament cross-linking and filament disassembly alone may suffice for cytokinesis, whereas in others such as *S. pombe*, cytokinesis may depend on motor activity-dependent and -independent functions of myosin II.

SUPPLEMENTAL INFORMATION

Supplemental Information includes Supplemental Experimental Procedures, three figures, and four movies and can be found with this article online at http://dx.doi.org/10.1016/j.cub.2017.01.028.

AUTHOR CONTRIBUTIONS

S.P. conceived and designed experiments, acquired data, performed analysis and interpretation of data, and drafted/revised the article. S.R. and M.M. generated yeast strains and performed preliminary analysis. T.G.C., A.K., S.H., B.C.L., M.S., and R.H. performed analysis and interpretation of data

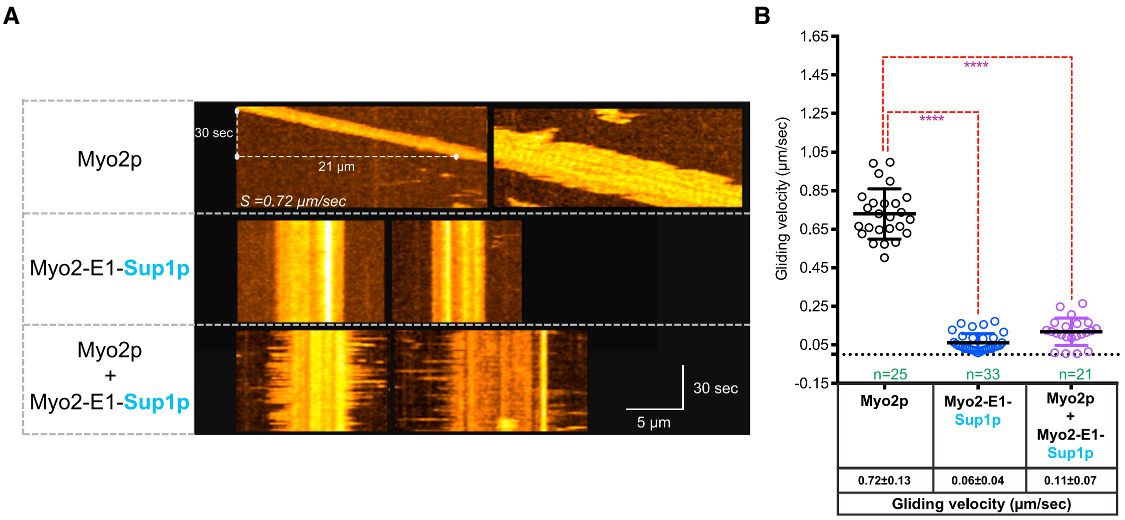

Figure 4. Myo2-E1-Sup1p Showed Tighter Actin Binding but No Motility

(A) Type II Myosin-based actin filament-gliding assay. Representative kymographs of time-lapse fluorescence micrographs of actin filaments labeled with rhodamine-phalloidin are shown. Scale bars represent 5 μm.

(B) Quantification of the actin filament-gliding assay of (A). Different myosins (Myo2p, Myo2-E1-Sup1p, and Myo2p + Myo2-E1-Sup1p) were tested for gliding velocity (μm/s) using rhodamine-phalloidin-labeled actin.

See also Figure S3 and Movies S1, S2, S3, and S4.

and generated yeast strains and reagents. P.G. performed structural analysis and interpretation of data and drafted/revised the article. M.K.B. conceived the project, conceived and designed experiments, and performed analysis and interpretation of data. S.P. and M.K.B. wrote the manuscript. All authors reviewed the manuscript.

ACKNOWLEDGMENTS

We thank Matt Lord, Kathy Trybus, and Luther Pollard for yeast strains and plasmids. Many thanks are due to members of the Balasubramanian laboratory for discussion and Rob Cross for critical comments. This work was funded by Warwick Medical School, Royal Society Wolfson Merit Award, and Wellcome Trust (WT101885MA). The early part of the work (described in Figure 1A) was performed in Temasek Life Sciences Laboratory, Singapore. P.G. acknowledges fellowships from INSPIRE, Department of Science and Technology, Government of India and an Innovative Young Biotechnologist Award (IYBA), Department of Biotechnology. S.H. acknowledges IISER Pune for a PhD fellowship. M.M. is an Intermediate Fellow of the Wellcome Trust-DBT India Alliance (IA/I/14/1/501317). M.M. acknowledges the India Alliance and the DAE/TIFR for funds.

Received: April 12, 2016
Revised: November 21, 2016
Accepted: January 16, 2017
Published: February 23, 2017

REFERENCES

1. Cheffings, T.H., Burroughs, N.J., and Balasubramanian, M.K. (2016). Actomyosin Ring Formation and Tension Generation in Eukaryotic Cytokinesis. Curr. Biol. *26*, R719–R737.
2. Pollard, T.D., and Wu, J.Q. (2010). Understanding cytokinesis: lessons from fission yeast. Nat. Rev. Mol. Cell Biol. *11*, 149–155.
3. Green, R.A., Paluch, E., and Oegema, K. (2012). Cytokinesis in animal cells. Annu. Rev. Cell Dev. Biol. *28*, 29–58.
4. Ma, X., Kovács, M., Conti, M.A., Wang, A., Zhang, Y., Sellers, J.R., and Adelstein, R.S. (2012). Nonmuscle myosin II exerts tension but does not translocate actin in vertebrate cytokinesis. Proc. Natl. Acad. Sci. USA *109*, 4509–4514.
5. Mendes Pinto, I., Rubinstein, B., Kucharavy, A., Unruh, J.R., and Li, R. (2012). Actin depolymerization drives actomyosin ring contraction during budding yeast cytokinesis. Dev. Cell *22*, 1247–1260.
6. Balasubramanian, M.K., McCollum, D., Chang, L., Wong, K.C., Naqvi, N.I., He, X., Sazer, S., and Gould, K.L. (1998). Isolation and characterization of new fission yeast cytokinesis mutants. Genetics *149*, 1265–1275.
7. Wong, K.C., Naqvi, N.I., Iino, Y., Yamamoto, M., and Balasubramanian, M.K. (2000). Fission yeast Rng3p: an UCS-domain protein that mediates myosin II assembly during cytokinesis. J. Cell Sci. *113*, 2421–2432.
8. Mishra, M., Kashiwazaki, J., Takagi, T., Srinivasan, R., Huang, Y., Balasubramanian, M.K., and Mabuchi, I. (2013). In vitro contraction of cytokinetic ring depends on myosin II but not on actin dynamics. Nat. Cell Biol. *15*, 853–859.
9. Kitayama, C., Sugimoto, A., and Yamamoto, M. (1997). Type II myosin heavy chain encoded by the myo2 gene composes the contractile ring during cytokinesis in Schizosaccharomyces pombe. J. Cell Biol. *137*, 1309–1319.
10. Stark, B.C., James, M.L., Pollard, L.W., Sirotkin, V., and Lord, M. (2013). UCS protein Rng3p is essential for myosin-II motor activity during cytokinesis in fission yeast. PLoS ONE *8*, e79593.
11. Lord, M., and Pollard, T.D. (2004). UCS protein Rng3p activates actin filament gliding by fission yeast myosin-II. J. Cell Biol. *167*, 315–325.
12. Morrell-Falvey, J.L., Ren, L., Feoktistova, A., Haese, G.D., and Gould, K.L. (2005). Cell wall remodeling at the fission yeast cell division site requires the Rho-GEF Rgf3p. J. Cell Sci. *118*, 5563–5573.
13. Davidson, R., Laporte, D., and Wu, J.Q. (2015). Regulation of Rho-GEF Rgf3 by the arrestin Art1 in fission yeast cytokinesis. Mol. Biol. Cell *26*, 453–466.
14. Laplante, C., Berro, J., Karatekin, E., Hernandez-Leyva, A., Lee, R., and Pollard, T.D. (2015). Three myosins contribute uniquely to the assembly and constriction of the fission yeast cytokinetic contractile ring. Curr. Biol. *25*, 1955–1965.

15. Bezanilla, M., Forsburg, S.L., and Pollard, T.D. (1997). Identification of a second myosin-II in Schizosaccharomyces pombe: Myp2p is conditionally required for cytokinesis. Mol. Biol. Cell *8*, 2693–2705.
16. Motegi, F., Nakano, K., Kitayama, C., Yamamoto, M., and Mabuchi, I. (1997). Identification of Myo3, a second type-II myosin heavy chain in the fission yeast Schizosaccharomyces pombe. FEBS Lett. *420*, 161–166.
17. Bezanilla, M., Wilson, J.M., and Pollard, T.D. (2000). Fission yeast myosin-II isoforms assemble into contractile rings at distinct times during mitosis. Curr. Biol. *10*, 397–400.
18. Huang, J., Mishra, M., Palani, S., Chew, T.G., and Balasubramanian, M.K. (2016). Isolation of cytokinetic actomyosin rings from Saccharomyces cerevisiae and Schizosaccharomyces pombe. Methods Mol. Biol. *1369*, 125–136.
19. Vavylonis, D., Wu, J.Q., Hao, S., O'Shaughnessy, B., and Pollard, T.D. (2008). Assembly mechanism of the contractile ring for cytokinesis by fission yeast. Science *319*, 97–100.
20. Wu, J.Q., Sirotkin, V., Kovar, D.R., Lord, M., Beltzner, C.C., Kuhn, J.R., and Pollard, T.D. (2006). Assembly of the cytokinetic contractile ring from a broad band of nodes in fission yeast. J. Cell Biol. *174*, 391–402.
21. Lord, M., Laves, E., and Pollard, T.D. (2005). Cytokinesis depends on the motor domains of myosin-II in fission yeast but not in budding yeast. Mol. Biol. Cell *16*, 5346–5355.

Current Biology, Volume *27*

Supplemental Information

Motor Activity Dependent and Independent Functions of Myosin II Contribute to Actomyosin Ring Assembly and Contraction in *Schizosaccharomyces pombe*

Saravanan Palani, Ting Gang Chew, Srinivasan Ramanujam, Anton Kamnev, Shrikant Harne, Bernardo Chapa-y-Lazo, Rebecca Hogg, Mayalagu Sevugan, Mithilesh Mishra, Pananghat Gayathri, and Mohan K. Balasubramanian

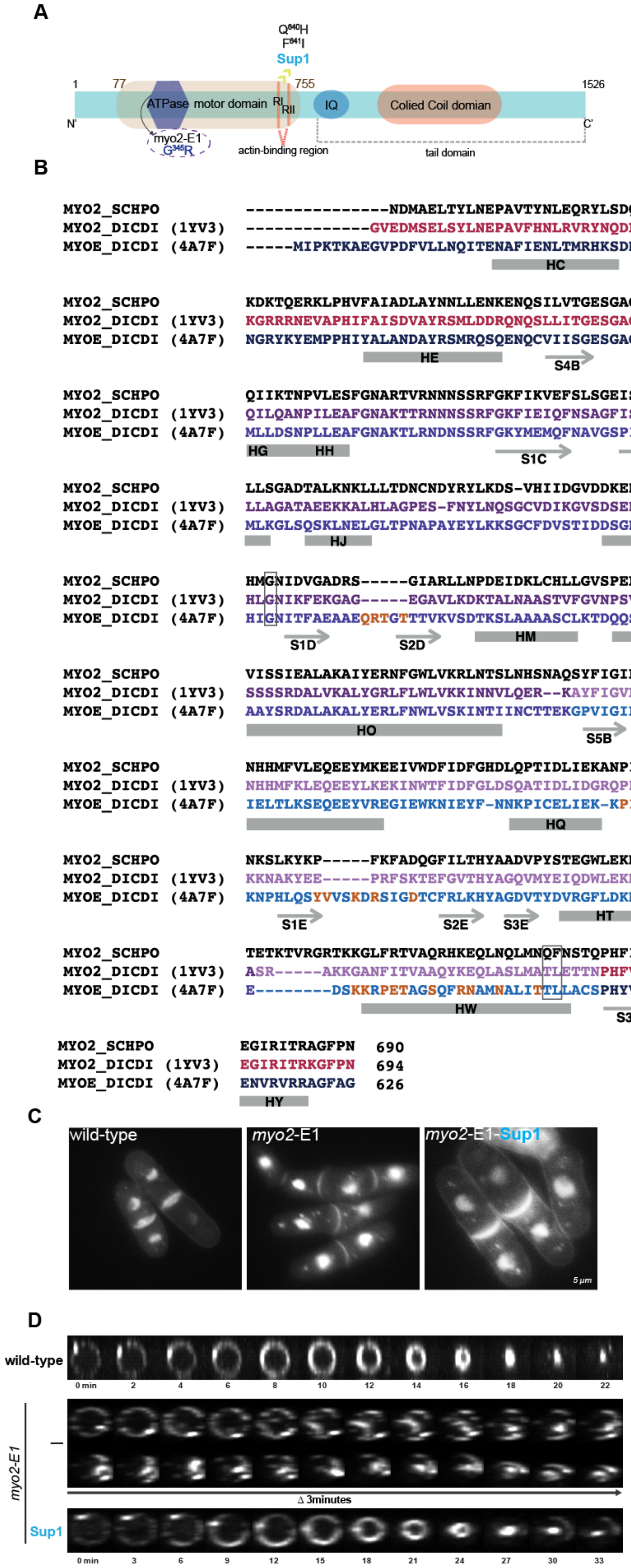
A
Q640H
F641I
Sup1
1
77
755
1526
ATPase motor domain
RI
RII
IQ
Colied Coil domian
N'
C'
myo2-E1
G345R
actin-binding region
tail domain
B
MYO2_SCHPO ----------------NDMAELTYLNEPAVTYNLEQRYLSDQIYTYSGLFLVAVNPYCGLPIYTKDIIQLY 134
MYO2_DICDI (1YV3) --------------GVEDMSELSYLNEPAVFHNLRVRYNQDLIYTYSGLFLVAVNPFKRIPIYTQEMVDIF 143
MYOE_DICDI (4A7F) -----MIPKTKAEGVPDFVLLNQITENAFIENLTMRHKSDNIYTYIGDVVISTNPFKNLNIYKESDIKAY 65
HC S1B S2B HD
MYO2_SCHPO KDKTQERKLPHVFAIADLAYNNLLENKENQSILVTGESGAGKTENTKRIIQYLAAIASSTTVGSSQVEE- 203
MYO2_DICDI (1YV3) KGRRRNEVAPHIFAISDVAYRSMLDDRQNQSLLITGESGAGKTENTKKVIQYLASVAGRnqangsgVLEQ 213
MYOE_DICDI (4A7F) NGRYKYEMPPHIYALANDAYRSMRQSQENQCVIISGESGAGKTEASKKIMQFLTFVSSNQSPNGERISK- 134
HE S4B HF
MYO2_SCHPO QIIKTNPVLESFGNARTVRNNNSSRFGKFIKVEFSLSGEISNAAIEWYLLEKSRVVHQNEFERNYHVFYQ 273
MYO2_DICDI (1YV3) QILQANPILEAFGNAKTTRNNNSSRFGKFIEIQFNSAGFISGASIQSYLLEKSRVVFQSETERNYHIFYQ 283
MYOE_DICDI (4A7F) MLLDSNPLLEAFGNAKTLRNDNSSRFGKYMEMQFNAVGSPIGGKITNYLLEKSRVVGRTQGERSFHIFYQ 204
HG HH S1C S6B S7B HI
MYO2_SCHPO LLSGADTALKNKLLLTDNCNDYRYLKDS-VHIIDGVDDKEEFKTLLAAFKTLGFDDKENFDLFNILSIIL 342
MYO2_DICDI (1YV3) LLAGATAEEKKALHLAGPES-FNYLNQSGCVDIKGVSDSEEFKITRQAMDIVGFSQEEQMSIFKIIAGIL 352
MYOE_DICDI (4A7F) MLKGLSQSKLNELGLTPNAPAYEYLKKSGCFDVSTIDDSGEFKIIVKAMETLGLKESDQNSIWRILAAIL 274
HJ HK HL
MYO2_SCHPO HMGNIDVGADRS-----GIARLLNPDEIDKLCHLLGVSPELFSQNLVRPRIKAG----HEWVISARSQTQ 403
MYO2_DICDI (1YV3) HLGNIKFEKGAG-----EGAVLKDKTALNAASTVFGVNPSVLEKALMEPRILAG----RDLVAQHLNVEK 413
MYOE_DICDI (4A7F) HIGNITFAEAAEQRTGTTTVKVSDTKSLAAAASCLKTDQQSLSIALCYRSISTGVGKRCSVISVPMDCNQ 344
S1D S2D HM HN S4D S3D
MYO2_SCHPO VISSIEALAKAIYERNFGWLVKRLNTSLNHSNAQSYFIGILDIAGFEIFEKNSFEQLCINYTNEKLQQFF 473
MYO2_DICDI (1YV3) SSSSRDALVKALYGRLFLWLVKKINNVLQER--KAYFIGVLDISGFEIFKVNSFEQLCINYTNEKLQQFF 482
MYOE_DICDI (4A7F) AAYSRDALAKALYERLFNWLVSKINTIINCTTEKGPVIGILDIYGFEVFQNNSFEQLNINFCNEKLQQLF 414
HO S5B HP
MYO2_SCHPO NHHMFVLEQEEYMKEEIVWDFIDFGHDLQPTIDLIEKANPIGILSCLDEECVMPKATDATFTSKLDALWR 543
MYO2_DICDI (1YV3) NHHMFKLEQEEYLKEKINWTFIDFGLDSQATIDLIDGRQPPGILALLDEQSVFPNATDNTLITKLHSHFS 552
MYOE_DICDI (4A7F) IELTLKSEQEEYVREGIEWKNIEYF-NNKPICELIEK-KPIGLISLLDEACLIAKSTDQTFLDSICKQFE 482
HQ HR HS
MYO2_SCHPO NKSLKYKP-----FKFADQGFILTHYAADVPYSTEGWLEKNTDPLNENVAKLLAQSTNKHVATLFSDYQE 608
MYO2_DICDI (1YV3) KKNAKYEE-----PRFSKTEFGVTHYAGQVMYEIQDWLEKNKDPLQQDLELCFKDSSDNVVTKLFNDPNI 617
MYOE_DICDI (4A7F) KNPHLQSYVVSKDRSIGDTCFRLKHYAGDVTYDVRGFLDKNKDTLFGDLISSMQSSSDPLVQGLFPPTRP 552
S1E S2E S3E HT HU HV
MYO2_SCHPO TETKTVRGRTKKGLFRTVAQRHKEQLNQLMNQFNSTQPHFIRCIVPNEEKKMHTFNRPLVLGQLRCNGVL 678
MYO2_DICDI (1YV3) ASR-----AKKGANFITVAAQYKEQLASLMATLETTNPHFVRCIIPNNKQLPAKLEDKVVLDQLRCNGVL 682
MYOE_DICDI (4A7F) E--------DSKKRPETAGSQFRNAMNALITTLLACSPHYVRCIKSNDNKQAGVIDEDRVRHQVRYLGLL 614
HW S3B HX
MYO2_SCHPO EGIRITRAGFPN 690
MYO2_DICDI (1YV3) EGIRITRKGFPN 694
MYOE_DICDI (4A7F) ENVRVRRAGFAG 626
HY
C
wild-type
myo2-E1
myo2-E1-Sup1
5 μm
D
wild-type
0 min
2
4
6
8
10
12
14
16
18
20
22
myo2-E1
Δ 3minutes
Sup1
0 min
3
6
9
12
15
18
21
24
27
30
33

Figure S1 (related to Figure 1): Schematic representation of myosin II mutants and 3D-projected rings from wild-type, *myo2*-E1 and *myo2*-E1-Sup1.

A. Schematic of type-II myosin (Myo2) domains. The *myo2*-E1 mutation site (G345R), and the intragenic suppressor site (*myo2*-E1-Sup1[Q640H; F641I]), identified via a UV based screen, are highlighted.
B. Multiple sequence alignment of S. *pombe* Myo2p, *Dictyostelium discoideum* Myo2p and MyoEp protein sequences. The *myo2*-E1 (G345R) and the intragenic suppressor *myo2*-E1-Sup1 are marked with boxes around them. The actin-interacting residues are highlighted in orange, while the domain organization is color coded as in Figure S2. The residues missing in the crystal structure are shown in lowercase letters.
C. Cells were grown at 24°C and shifted for 4-6h at 36°C before PFA fixation. DAPI and anillin blue staining used to visualize the nucleus and septum of wild type, *myo2*-E1 and *myo2*-E1-Sup1 intragenic suppressors cells respectively.
D. Time-series of 3D projected end-on view of actomyosin rings from wild-type, *myo2*-E1 and *myo2*-E1-Sup1 cells. Scale bars represent 3 µm.

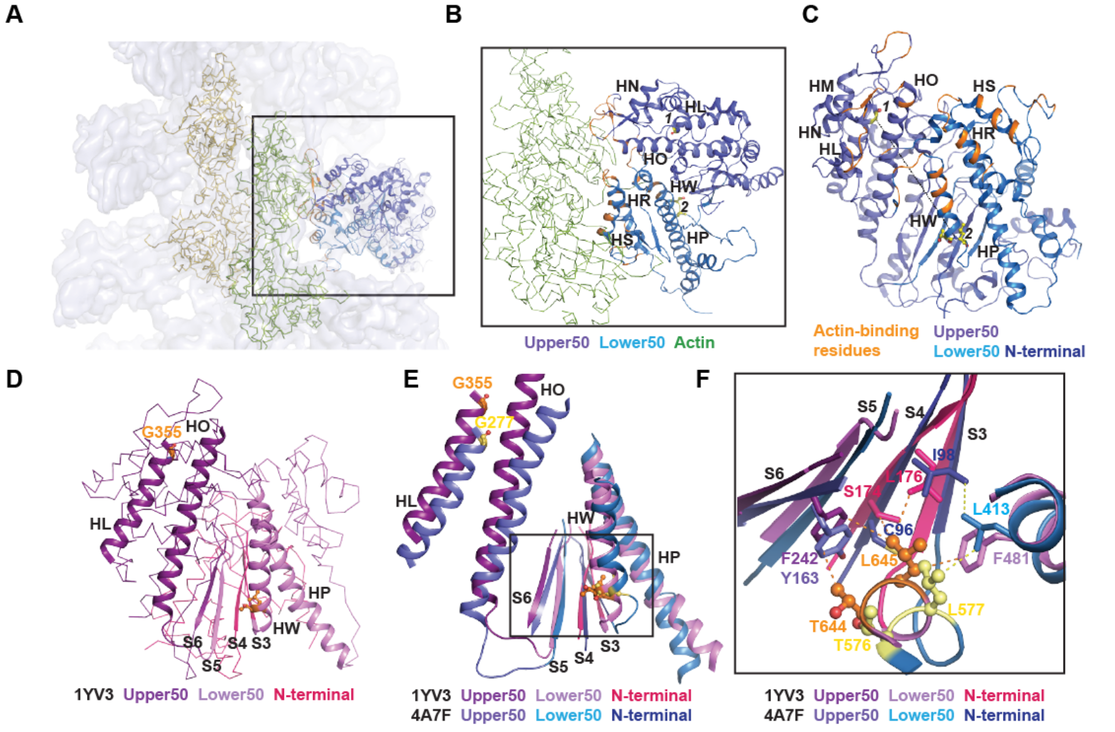

Figure S2 (related to Figure 2 and 3): Analysis of available myosin II and actin structures provides insight into the ring-contraction defect of *myo2*-E1-Sup1.

A. Cryoelectron microscopy reconstruction of a section of actomyosin structure (EMD-1987; PDB ID 4A7F). The subunits in the two protofilaments of actin are shown in yellow and green, while one of the myosin heads is shown in cartoon representation (refer to Supplementary Figure 1B for details of color coding of domains and secondary structure labeling). The residues at the actin-binding interface are highlighted in orange.
B. Zoomed-in view of the actin-myosin interface with the key secondary structure elements labeled. Residues corresponding to G345 (*myo2*-E1) and Q640-F641 (*myo2*-E1-Sup1) are highlighted in stick representation in yellow (labeled as 1 and 2 respectively).
C. The actin-binding interface of myosin involves residues from the Upper 50-K and Lower 50-K sub-domains, and hence relative movement between the two sub-domains contributes to binding efficiency. The distance between *myo2*-E1 and *myo2*-E1-Sup1 is highlighted by the dashed line and corresponds to 36 Å. It is to be noted that the side chains of Q640H and F641I point away from the actin-binding interface.

D. Key secondary structure elements of the myosin Upper 50-K and Lower 50-K sub-domains that undergo conformational changes during the cross-bridge cycle of myosin are highlighted in cartoon representation in a ribbon model of Dictyostelium discoideum myosin II (1YV3). Except for strand S4 of the transducer sheet, the N-terminal domain is not shown for clarity.
E. Key secondary structure elements in (D) are shown. The location of *myo2*-E1 (labeled as G355 and G277 in 1YV3 and 4A7F, respectively) and *myo2*-E1-Sup1 (shown in stick representation on helix HW) are highlighted.
F. Zoomed view of the box in (E) highlights the neighbouring residues of *myo2*-E1-Sup1. The residues T644 and L645 in 1YV3, and T576 and L577 in 4A7F structures correspond to Q640 and F641 of S. pombe Myo2.

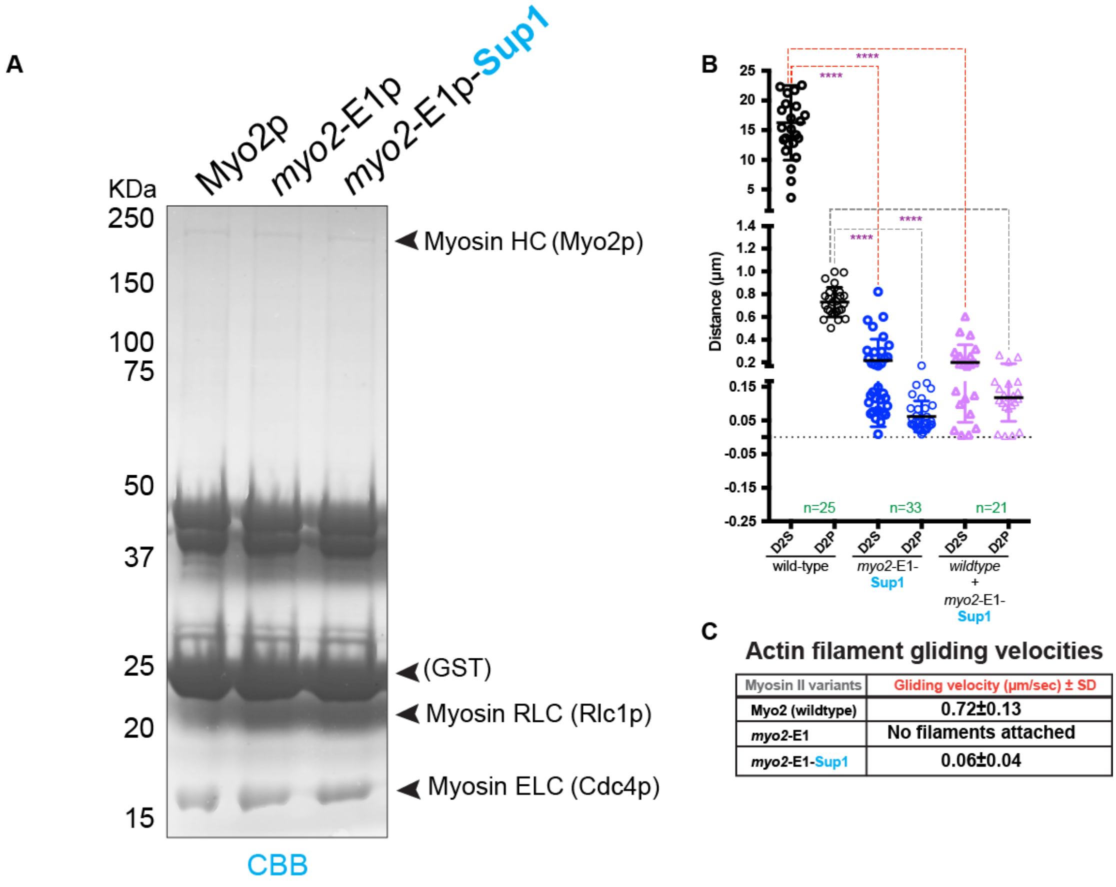

Myosin II variants	Gliding velocity (μm/sec) ± SD
Myo2 (wildtype)	0.72±0.13
myo2-E1	No filaments attached
myo2-E1-Sup1	0.06±0.04

Figure S3 (related to Figure 4): Myosin purification and gliding assay.

A. SDS-PAGE of purified Type-II myosin complex (Myo2p/Cdc4p/Rlc1p) proteins stained with Coomassie brilliant blue (CBB, SimplyBlue Safestain, Invitrogen). Three lanes show samples from three different myosin complex variants with their light chains. All of them were co-overexpressed using the *nmt41* thiamine repressible promoter for 28-36 hr at 24°C. One step purified myosin complexes (1 mg/ml) were obtained after removal of the GST tag from the light chains.
B. Quantification of the actin filament gliding velocities of Figure 4A. D2S (distance from the initial point of the track to the current point) and D2P (distance from the current point to the preceding point of the track) were plotted separately for the indicated purified myosin variants (Myo2p, myo2-E1p-Sup1, Myo2p+myo2-E1p-Sup1).
C. Actin filament gliding velocities were calculated from different myosin variants (n > 20 filaments per sample).

Supplemental experimental procedures

Yeast genetics and culture methods:

Cells were grown and cultured in Yeast Extract Medium (YES). For the selection of plasmids, cells were grown in Edinburgh Minimal Medium (EMM) with appropriate supplements, as previously described [S1]. ELN (Extremely Low Nitrogen) plates have the same composition as EMM, except that NH_4Cl (50mg/liter) were mainly used for mating and sporulation.

Screen for intragenic suppressors of *myo2*-E1:

Log phase *myo2*-E1 clp1Δ cells plated on YEA agar plates (~2000 cells/plate) were exposed to UV light (9000 μJ/cm^2) for 6 seconds in a UV cross linker (UVP CL-1000 UV cross linker). Plates were stored in the dark at 24°C after UV exposure for 16-20 hours prior to shifting the plates to 36°C for 10-12 days. Positive/rescuing colonies were grown on YEA plates after the 10-12 days of growth at 36°C. Colony PCR was performed on the colonies grown at 36°C using myo2 primers and the PCR products were sequenced to identify the intragenic suppressor of *myo2*-E1.

Fluorescence microscopy:

Cells were grown at 24°C in Yeast Extract Medium (YES) to mid-log phase and shifted to 36°C for 6 hours before fixation. For visualization of DAPI and anillin blue staining, cells were fixed with 4% paraformaldehyde (PFA) and permeabilised with 1% Triton-X-100 at room temperature for 10 minutes. Cells were washed thrice with 1X PBS without detergent and stained with DAPI to visualize DNA and anillin blue to visualize septa and cell wall. Still images were acquired using a wide-field fluorescent microscope (Andor Revolution wide-field imaging system, Andor), equipped with a 100x oil immersion 1.49NA Nikon TIRF Apo objective, an Andor sCMOS ZYLA detector, and Andor iQ software). More than 250 cells were counted per time point. Imaging software ImageJ or Fiji was used to process the images. Where appropriate, statistical significance was determined using Student's t-test (****P<0.0001). PRISM 6.0 software (GraphPad) was used for quantification (s.d. = standard deviation).

Live cell imaging:

For time-lapse live cell imaging, mid log phase cells were grown at 24°C and shifted to 36°C for 4-5 hours prior to imaging. Time-lapse movies were acquired for 3-4 hours under a fully controlled 36°C incubation chamber. During imaging, the cells were kept in CellASIC microfluidic yeast plates (Y04C and D size) or in Ibidi microscopic small chambers using a mineral oil suspension method. Time-lapse series were acquired using a spinning disk confocal microscope (Andor Revolution XD imaging system, equipped with a 100x oil immersion 1.45NA Nikon Plan Apo lambda objective, a Confocal Yokogawa CSU-X1 unit, an Andor sCMOS ZYLA detector, and Andor iQ software). When using Cell ASIC plates, Z-stacks comprised of fifteen 0.5 μm

spaced slices were generated for *Rlc1*-3GFP and mCh-atb2 at 1 minute intervals.

Cell ghost preparation and ATP treatment:

Cells were grown at 24°C on minimal medium and rings were isolated as previously described [S2, S3]. Cell ghosts were prepared from wild-type, *myo2*-E1, *myp2*Δ, *myo2*-E1 *myp2*Δ, *myo2*-E1-Sup1 and *myo2*-E1-Sup1 *myp2*Δ cells. *Rlc1*-3GFP was used as a ring marker and images shown are maximum intensity projections of z-stacks. Experiments were done at 24°C. Cell ghosts from the wild-type and mutants were treated with 0.5 mM ATP (t=0) and images were acquired every 30 seconds for 5 minutes. Images were processed using Andor iQ and Fiji imaging software.

Protein Purification:

Three individual strains, wild-type myosin Myo2p (MLP 509), motor defective myosin myo2-E1p (MLP469), and a myosin suppressor allele myo2-E1p-Sup1 (MBY11070), all overexpressing Myo2p from a thiamine controlled expression system under control of the full strength *nmt41* promoter, were co-transformed with GST-tagged light chains from the plasmids pGST-cdc4 and pGST-rlc1. Protein purification, GST tag cleavage, and storage of all myosins, together with their light chains, were performed as previously described [S4]. We used a similar method for the one-step purification of crude myosin. One-step purified myosin protein concentration was determined by Bradford assay with BSA as the standard. Purified myosins were resolved in 4-20% SDS-PAGE gradient gels and stained with Coomassie Brilliant Blue (CBB, SimplyBlue Safe stain, Invitrogen).

In vitro motility assay:

We performed the motility assay using the one step purified myosin and rhodamine-phalloidin stabilised actin filaments as previously described [S4, S5]. One step purified myosin was used at 0.25 µg/µl (total crude protein). Dilution buffer was used to normalise the protein concentration of all myosin variants (myo2-E1p and myo2-E1p-Sup1). Motility of the actin filaments was visualised by spinning disk confocal microscopy (Andor Revolution XD imaging system, equipped with a 100x oil immersion 1.45NA Nikon Plan Apo lambda objective, a Confocal Yokogawa CSU-X1 unit, an Andor sCMOS ZYLA detector, and Andor iQ software) and recorded at one second intervals. Movies were processed using FIJI (ImageJ) and the MTrackJ plug-in [S6]. Individual filament gliding velocities were quantified (n > 20 filaments).

In vitro gliding assay kymographs:

First, filament movement trajectories were visualized through a SUM projection of 20-30 frames of the raw time-lapse images (corresponding to a 20-30 seconds time interval). Next, a linear ROI was manually drawn along the trajectory of the chosen filament. Finally, a kymograph of filament displacement was created with the KymoResliceWide plugin in Fiji (http://imagej.net/KymoResliceWide) using the original time-lapse image and the previously defined ROI with a line thickness of 1.5 µm.

List of strains used in this study

MBY192	*ura4*-D18, *leu1*-32, h-	Lab stock
MBY8841	mCherry-*atb2*::hph; *rlc1*-3GFP::kanMx6; ura4-D18 ade6-210 leu1-32 h+	This study
MBY977	*clp1*::Ura4+, *ura4*-D18 *leu1*-32 *ade6*-21X h+	[S14]
MBY151	*myo2*-E1 *ade6*-21x *ura4*-D18 *leu1*-32 *his3*-d h-	[S15]
MBY2117	*myo2*-E1 *clp1*::*ura4*+ *ade6*-21X *ura4*-D18 *leu1*-32	[S16]
MBY10024	*myo2*-E1[G345R] mCherry-*atb2*::hph; *rlc1*-3*GFP*::*kanMx6*; *ura4* -D18 *ade6*-210 *leu1*-32	This study
MBY8932	*myo2*-E1[G345R]-Sup1[Q640H-F641I] mCherry-atb2::hph; *rlc1-3GFP::kanMx6*; *ura4* -D18 *ade6*-210 *leu1*-32 h-	This study
MBY10075	*myp2*::natMX6 mCherry-*atb2::hph*; *rlc1-3GFP::KanMX6* *ade6*-21X	This study
MBY10077	*myp2*::natMX6 *myo2*-E1 mCherry-*atb2*::hph, rlc1-3GFP::KanMX6 ade6-21	This study
MBY10085	*myp2*::natMX6 *myo2*-E1[G345R]-Sup1[Q640H-F641I] mCherry-atb2::hph; Rlc1-3GFP::KanMX6 ade6-21X	This study
MLP 509	leu1-32 ura4::kanR natR:41nmt1prom-*myo2* h-	[S4]
MLP 469	leu1-32 his7-366 ura4-D18 ade6-M216 natR:41nmt1prom-*myo2*-E1 h-	[S4]
MBY11070	leu1-32 his7-366 natR:41nmt1prom-*myo2*-E1-Sup1 (Q640H F641I) h?	This study
MBY11074	pDS472-URA4-Rlc1; pDS473-LEU2-Cdc4 was transformed in to MLP 509 (natR::41nmt1prom-*myo2*) h-	This study
MBY11075	pDS472-URA4-Rlc1; pDS473-LEU2-Cdc4 was transformed in to MLP 469 (natR::41nmt1prom-*myo2*-E1) h-	This study
MBY11076	pDS472-URA4-Rlc1; pDS473-LEU2-Cdc4 was transformed in to MBY11070 (natR::41nmt1prom-*myo2*-E1-Sup1 [Q640H; F641I]) h?	This study

Structure analysis and illustration:

The myosin structures were downloaded from PDB (Protein Data Bank) and EMDB (Electron microscopy database). Structural analysis and illustrations were carried out using PyMOL (Schrodinger). Domain wise structural superpositions were performed for observing relative domain movements. The multiple sequence alignment shown in the Supplementary Figure S1B was obtained using PROMALS3D [S7].

In Myo2-E1p (product of myo2-E1), the bulky side chain of arginine in the place of glycine (G345R) constrains the relative movements between helices HO and HL of the upper 50 kDa sub-domain (Figure S2). This in turn may restrict the interdomain movements between the upper 50 kDa and lower 50 kDa sub-domains, which are essential for actin binding and ATP hydrolysis by Myo2p (Figure S2A-C). The myo2-E1-Sup1 mutations Q640H and F641I are located towards the C-terminal end of the helix HW of the lower 50 kDa sub-domain (Figure S2), far apart from G345 (~36 Å, Figure S2C). These residues are not directly located at the actin-binding interface or near the ATP-binding site. Figure S2A-C highlights the relative position of the mutations and the actin-binding interface of myosin (PDB ID 4A7F; EMD-1987; [S8]) .

Domain-wise structural superposition of various conformations of published myosin crystal structures demonstrates that helices HL (which contains G345), HO in the upper 50 kDa sub-domain, strands 3-6 of the transducer sheet, helix HW, and helix HP (relay helix) undergo key coupled conformational changes (Figure S2D), thus playing a significant role in the allosteric changes during the conformational cycle of myosin. Shown in Figure S2 E and F is the superposition of the key secondary structures mentioned above on the structures of actin-bound myosin IE (PDB ID 4A7F and EMDB ID EMD-1987; [S8]) and myosin II bound to blebbistatin and an ATP analogue (PDB ID 1YV3; [S9]) from Dictyostelium discoideum. The structures were chosen as representatives of the actin-bound and unbound conformations of myosin. Helices HL and HO in the upper 50 kDa sub-domain undergo concerted movement. The conformational movement in the upper 50 kDa sub-domain is coupled to changes in the twist of the transducer beta-sheet, and a kink formation within the relay helix (HP) during the myosin conformational cycle [S10-S12]. Analysis of the crystal structures shows that residues equivalent to Q640 and F641 occupy key positions that could potentially affect these conformational transitions. The equivalent residues corresponding to the F641 side chain (L577/L645 in Figure S2) interact with the residues at the kink of helix HP, and that of Q640 with residues that interact with the transducer sheet (T576/T644 in Figure S2). The kinking movement of the relay helix is crucial in coupling the actin-binding and ATP hydrolysis states of myosin with the conformations of the converter domain and the lever arm. Thus, changes at the position corresponding to Q640 and F641 in myosin might rescue its ability to bind to actin by relieving the constraints imposed by the G345R mutation and promoting a rigor-like state with increased affinity for F-actin, leading to reduced contraction. However, the mutations in Myo2E1-Sup1 appear to have resulted in an increased

affinity for actin due to the increased hydrophobic interactions of the lower 50 kDa sub-domain with the transducer sheet and the relay helix. This increase in affinity to actin thereby led to the defects observed in the contraction of the actomyosin ring.

Well-characterized mutations that affect the coupling between actin-binding and ATP-hydrolysis with the conformations of the converter domain and the lever arm, and thereby with translocation, are the R703C and N93K mutations [S13]. Residues equivalent to R703 and N93 are also implicated in influencing the position of the relay helix in response to the ATP state of myosin. Analysis of R703 equivalent residues in myosin crystal structures show that it communicates to the N-terminal domain (where ATP hydrolysis takes place) through main chain interactions with N93 [S13]. This dictates the position of helix HY, where R703 is located, and subsequently the relay helix and the converter. Similarly, the location of the mutations in myo2-E1-Sup1 mutants reported in this work also communicates to the N-terminal domain through interactions with the strands of the transducer sheet and the relay helix through the hydrophobic patch at its kink. Hence, it is probable that Myo2-E1-Sup1p may function similarly to these R703C and N93K mutants.

Supplemental References:

S1. Moreno, S., Klar, A., and Nurse, P. (1991). Molecular genetic analysis of fission yeast Schizosaccharomyces pombe. Methods Enzymol *194*, 795-823.

S2. Huang, J., Mishra, M., Palani, S., Chew, T.G., and Balasubramanian, M.K. (2016). Isolation of Cytokinetic Actomyosin Rings from Saccharomyces cerevisiae and Schizosaccharomyces pombe. Methods Mol Biol *1369*, 125-136.

S3. Mishra, M., Kashiwazaki, J., Takagi, T., Srinivasan, R., Huang, Y., Balasubramanian, M.K., and Mabuchi, I. (2013). In vitro contraction of cytokinetic ring depends on myosin II but not on actin dynamics. Nat Cell Biol *15*, 853-859.

S4. Lord, M., and Pollard, T.D. (2004). UCS protein Rng3p activates actin filament gliding by fission yeast myosin-II. J Cell Biol *167*, 315-325.

S5. Tang, Q., Pollard, L.W., and Lord, M. (2016). Measurements of Myosin-II Motor Activity During Cytokinesis in Fission Yeast. Methods Mol Biol *1369*, 137-150.

S6. Meijering, E., Dzyubachyk, O., and Smal, I. (2012). Methods for cell and particle tracking. Methods Enzymol *504*, 183-200.

S7. Pei, J., Kim, B.H., and Grishin, N.V. (2008). PROMALS3D: a tool for multiple protein sequence and structure alignments. Nucleic Acids Res *36*, 2295-2300.

S8. Behrmann, E., Muller, M., Penczek, P.A., Mannherz, H.G., Manstein, D.J., and Raunser, S. (2012). Structure of the rigor actin-tropomyosin-myosin complex. Cell *150*, 327-338.

S9. Allingham, J.S., Smith, R., and Rayment, I. (2005). The structural basis of blebbistatin inhibition and specificity for myosin II. Nat Struct Mol Biol *12*, 378-379.

S10. Yang, Y., Gourinath, S., Kovacs, M., Nyitray, L., Reutzel, R., Himmel, D.M., O'Neall-Hennessey, E., Reshetnikova, L., Szent-Gyorgyi, A.G., Brown, J.H., et al. (2007). Rigor-like structures from muscle myosins reveal key mechanical elements in the transduction pathways of this allosteric motor. Structure *15*, 553-564.

S11. Sweeney, H.L., and Houdusse, A. (2010). Structural and functional insights into the Myosin motor mechanism. Annu Rev Biophys *39*, 539-557.

S12. Preller, M., and Manstein, D.J. (2013). Myosin structure, allostery, and mechano-chemistry. Structure *21*, 1911-1922.

S13. Kim, K.Y., Kovacs, M., Kawamoto, S., Sellers, J.R., and Adelstein, R.S. (2005). Disease-associated mutations and alternative splicing alter the enzymatic and motile activity of nonmuscle myosins II-B and II-C. J Biol Chem *280*, 22769-22775.

S14. Trautmann, S., Wolfe, B.A., Jorgensen, P., Tyers, M., Gould, K.L., and McCollum, D. (2001). Fission yeast Clp1p phosphatase regulates G2/M transition and coordination of cytokinesis with cell cycle progression. Curr Biol *11*, 931-940.

S15. Balasubramanian, M.K., McCollum, D., Chang, L., Wong, K.C., Naqvi, N.I., He, X., Sazer, S., and Gould, K.L. (1998). Isolation and

characterization of new fission yeast cytokinesis mutants. Genetics *149*, 1265-1275.

S16. Mishra, M., Karagiannis, J., Trautmann, S., Wang, H., McCollum, D., and Balasubramanian, M.K. (2004). The Clp1p/Flp1p phosphatase ensures completion of cytokinesis in response to minor perturbation of the cell division machinery in Schizosaccharomyces pombe. J Cell Sci *117*, 3897-3910.
